# Celastrol: A Review of Useful Strategies Overcoming its Limitation in Anticancer Application

**DOI:** 10.3389/fphar.2020.558741

**Published:** 2020-11-18

**Authors:** Jinfeng Shi, Jiaxin Li, Ziyi Xu, Liang Chen, Ruifeng Luo, Chen Zhang, Fei Gao, Jinming Zhang, Chaomei Fu

**Affiliations:** State Key Laboratory of Southwestern Chinese Medicine Resources, Pharmacy School, Chengdu University of Traditional Chinese Medicine, Chengdu, China

**Keywords:** celastrol, anticancer activity, combination therapy, analogs of celastrol, nano/micro-formulation

## Abstract

Celastrol, a natural bioactive ingredient derived from *Tripterygium wilfordii* Hook F, exhibits significant broad-spectrum anticancer activities for the treatment of a variety of cancers including liver cancer, breast cancer, prostate tumor, multiple myeloma, glioma, etc. However, the poor water stability, low bioavailability, narrow therapeutic window, and undesired side effects greatly limit its clinical application. To address this issue, some strategies were employed to improve the anticancer efficacy and reduce the side-effects of celastrol. The present review comprehensively focuses on the various challenges associated with the anticancer efficiency and drug delivery of celastrol, and the useful approaches including combination therapy, structural derivatives and nano/micro-systems development. The specific advantages for the use of celastrol mediated by these strategies are presented. Moreover, the challenges and future research directions are also discussed. Based on this review, it would provide a reference to develop a natural anticancer compound for cancer treatment.

## Introduction

In the past few decades, the interest in natural medicinal plants and their bioactive molecules has extensively grown, and many of such agents exhibit diverse effects on suppressing the development and progression of tumor, both *in vitro* as well *in vivo* (Li, J. et al., 2019; Lagoa et al., 2020; Weng and Goel, 2020). It is widely believed that such substances can provide a plausible alternative to synthetic drugs due to their potency, safety, and lower cost (Hansen et al., 2011). One representative example is celastrol, also named tripterine, which is isolated from the *Tripterygium wilfordii* Hook F (TWHF) plant, also known as Lei Gong Teng (Thunder God Vine) (Landewé and van der Heijde, 2014). Celastrol is the most abundant and promising bioactive compound derived from TWHF (Corson and Crews, 2007; Liu et al., 2015; Chang et al., 2018), which is a quinone methide triterpenoid and has been voted as one of the top-five promising natural medicine molecules (Li and Hao 2019). Celastrol has been found to bear anticancer activities against sundry tumors as evidenced by promising results in multiple preclinical studies, including liver cancer, breast cancer, prostate cancer, leukemia, melanoma, etc. (Kashyap et al., 2018; Yadav et al., 2018).

**FIGURE 1:**
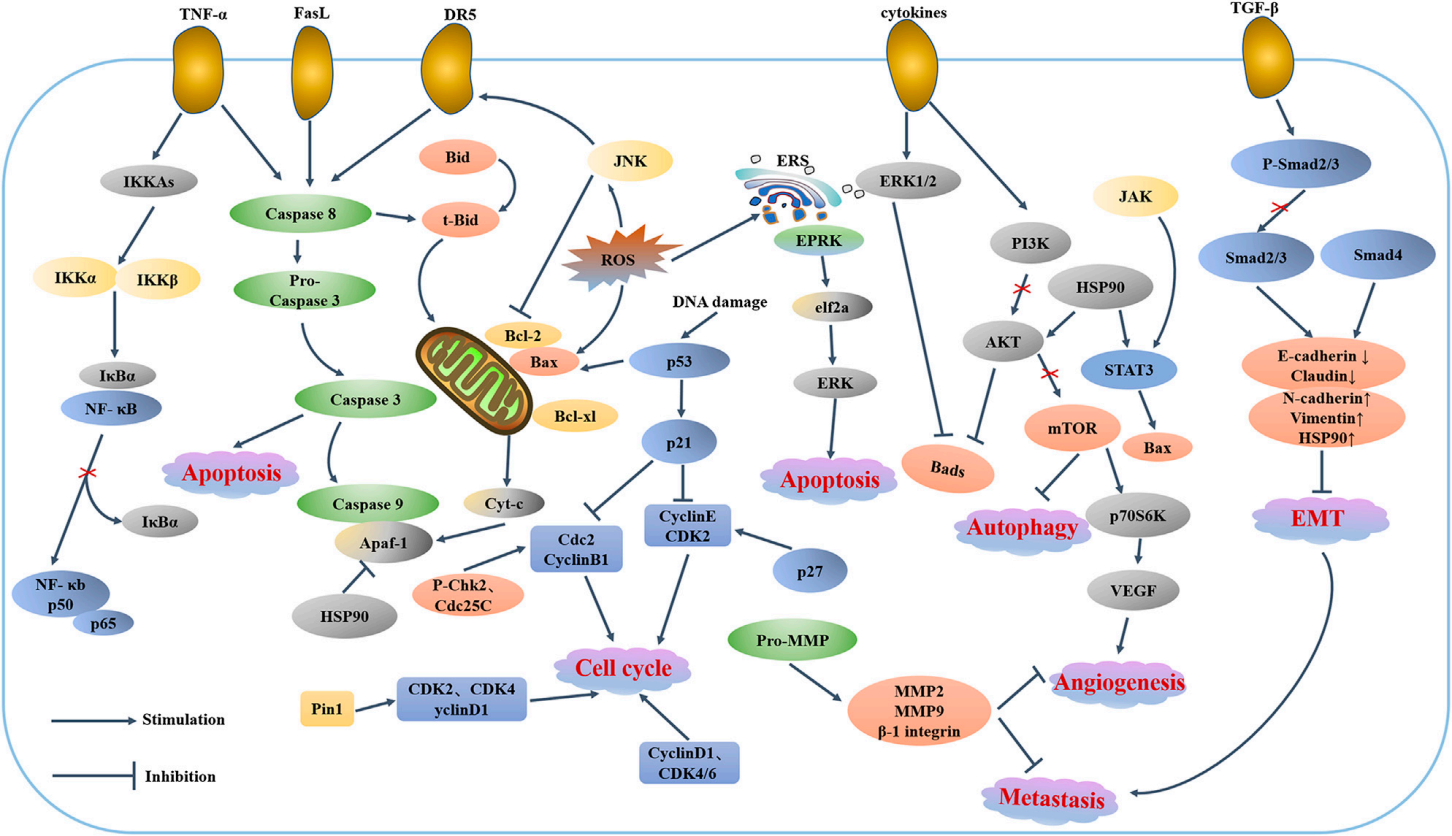
Celastrol-regulated cellular signaling pathways.

Recently, data from diﬀerent tumor cell lines and animal cancer models have suggested that the anticancer properties of celastrol can be attributed to: i) induced apoptosis and autophagy, ii) cell cycle arrest, iii) antimetastatic and anti-angiogenic actions, iv) anti-inflammatory effects, and Ⅴ) antioxidant activities (Cascão et al., 2017; Kashyap et al., 2018). It could target multiple signaling pathways such as reactive oxygen species (ROS)/JNK and Akt/mTOR (Liu et al., 2019), NF-κb (Chiang et al., 2014), STAT3/JAK2 (Rajendran et al., 2012), HSP90 (Sreeramulu et al., 2009; Zhang et al., 2009), Cdc37, p23, Iκκb, p-Akt (Kannaiyan et al., 2011), ERα (Jang et al., 2011), etc. In addition, the possible role of celastrol in the epithelial-mesenchymal transition (EMT) has aroused more and more concern (Avila-Carrasco et al., 2019). It has been discovered that celastrol could suppress EMT through up-regulating E-cadherin and down-regulating N-cadherin, Vimentin and Snail (Lin et al., 2015; Divya et al., 2018; Wang L.-P. et al., 2019). The Figure 1 lists some of the key signaling pathway proteins. All these properties make celastrol a promising drug for clinical application of anti-tumor drugs.

Despite the promising anticancer activities of celastrol, the clinical application of celastrol is strictly limited by severe side effects, mainly resulting from its undesirable bio-distribution, as well as various physicochemical and pharmacokinetic limitations, such as low water solubility and bioavailability (Wang et al., 2015; Li et al., 2020). Therefore, celastrol have been investigated to minimize or overcome these limitations (Maysinger et al., 2018; Hu et al., 2019). Raja et al. (2011) designed celastrol in combination with ErbB2-targeted therapeutics for treatment of ErbB2-overexpressing breast cancers. The results indicated celastrol significantly retarded the rate of growth of ErbB2-overexpressing human breast cancer cells in a mouse xenograft model with only minor systemic toxicity. Additionally, some researchers developed water-soluble analogs of celastrol for the translational development of celastrol. NST001A, a sodium salt of celastrol, inhibits the growth of human colon cancer cell-Colo 205 colon cells *in vitro* and *in vivo* (Tang et al., 2014). Furthermore, celastrol-albumin nanoparticles reduce the accumulation of free celastrol in off-target organs and tissues, thus successfully reducing the systemic toxicity of celastrol (Guo et al., 2017). Hence, celastrol has attracted considerable interests in developing new strategies to improve the anticancer efficacy and reduce the side-effects.

The aims of the present review are to summarize and critically analyze the recent development of different novel approaches for use of celastrol that are under cells and animal investigation in regards to minimize or overcome these limitations, as reported in the literature. The useful approaches including combination therapy, structural derivatives and nano/micro-systems development are discussed. The specific advantages for the use of celastrol mediated by these strategies are presented. Moreover, the challenges and future research directions are also discussed. Based on this review, it would provide a reference to develop a natural anticancer compound for cancer treatment.

### Limitations Associated With Celastrol Formulation Development

Although celastrol is very effective in treatment of many types of tumors, and could interact with many cellular targets, it also suffers some limitations such as poor water stability, low bioavailability, narrow therapeutic window, and undesired side effects (Cascão et al., 2017; Hou et al., 2020). These limitations have greatly hindered its clinical application.

Like so many chemotherapy drugs, celastrol has poor water solubility, which is 13.25 ± 0.83 mg/ml at 37°C (Qi et al., 2014). Owing to celastrol’s poor aqueous solubility, polyoxyethylated castor oil solvent (Cremophor) and ethanol may be used as vehicles. However, it would produce the toxicities associated with Cremophor, etc. (Hoy 2014). Besides, a study from Zhang et al. have demonstrated that oral administration of celastrol in rats results in ineﬀective absorption into the systemic circulation, with an absolute bioavailability of 17.06% (Zhang et al., 2012). Li Y. et al. (2012) suggest that besides low aqueous solubility *in vivo* metabolism and/or tissue distribution might also cause this poor bioavailability.

One great concern regarding the clinical use of celastrol is its narrow therapeutic window of dose together with the occurrence of adverse eﬀects. The effective dose of celastrol against various tumor xenograft model are reported to be around 3–5 mg/kg (Yang et al., 2006; Zhou and Huang, 2009; Chadalapaka et al., 2012; Liu et al., 2014; Wu et al., 2017). However, lower concentrations immediately lose efficacy and higher concentrations show signs of toxicity (Cascão et al., 2017). Similarly, in an osteosarcoma xenograft mouse model, it was described that treatment with celastrol at 1 and 2 mg/kg reduced tumor growth (42.9–50.2%), but it caused 5.7–9% weight loss in animals (Li et al., 2015). Discrepancies exist in celastrol dosing and toxicity, with data in rodents showing that at 3 mg/kg there are adverse events and 27% mortality but other studies showing no toxic eﬀects at this dose. In addition, there are reports showing an LD_50_ dose of 20.5 mg/kg and others suggesting a 40% mortality at 4 mg/kg (Cleren et al., 2005; Yang et al., 2006; Raja et al., 2011; Li et al., 2013; Konieczny et al., 2014). All of these results indicated that the therapeutic window of celastrol is very narrow. In addition, there are some side effects associated with celastrol, including infertility (Yuan et al., 1995; Bai et al., 2003), cardiotoxicity (Sun et al., 2006; Liu C. et al., 2019), hepatotoxicity (Sun et al., 2014; Jin et al., 2019), hematopoietic system toxicity (Lipsky and Tao, 1997; Kusy et al., 2012) and nephrotoxicity (Wu et al., 2018). Thus, much more attention should be paid for rational use of celastrol and its related preparations. Especially, the possibility of celastrol-drug interaction should be concerned significantly at the therapeutic concentrations.

### Strategies for the Use of Celastrol

**FIGURE 2:**
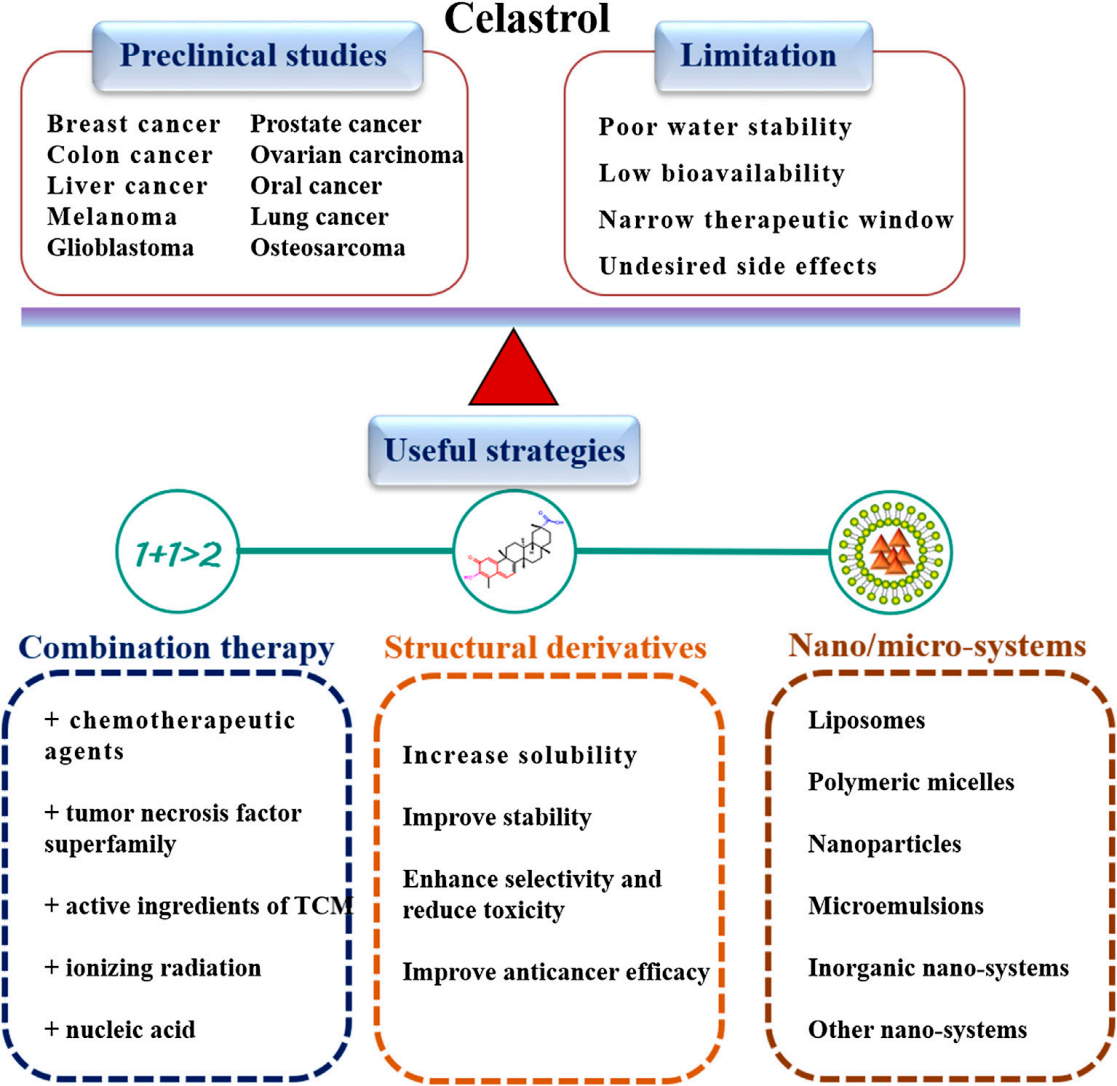
Different type of cancers that celastrol is found to manage. However, the poor water stability, low bioavailability, narrow therapeutic window, and undesired side effects greatly limit its clinical application. To address this issue, some strategies were employed to improve the anticancer efficacy and reduce the side-effects of celastrol.

To surpass the physicochemical and pharmacokinetic limitations of celastrol and to diminish the eﬀective dose, several methodologies have been tested that can represent useful strategies. Considerable effort has been exerted for the use of celastrol, including combination therapy, structural derivatives of celastrol and nano/micro-systems development (Figure 2).

### Combination Chemotherapy in Cancer Treatment

For a rational design to achieve optimal efficacy and reduce their toxicity, combination strategies used are essential (El Chediak et al., 2017; Gulijk et al., 2018). Lowering the dosage used of celastrol by combining it with agents effectively reduces its related adverse effects. Combination treatment offer opportunities for the translational development of celastrol. At present, the combination therapy of celastrol has been applied to the treatment of various types of cancer. Summary of the antitumor effects of celastrol and agents *in vitro* and *in vivo* are listed in Tables 1, 2.

**TABLE 1:** Summary of the antitumor effects of celastrol and chemotherapeutic drugs *in vitro*.

Combined treatment	Type of cancer	Cancer cell lines	Pathway	Findings	Ref
Lapatinib	Breast cancer	MDA-MB-453	Caspase-9 and Caspase-3↑; HER2, P-HER2, p-Akt, p-ERK1/2↓; HER2 membrane protein expression↓	Produce strong synergy in growth inhibition and apoptosis	(Yan et al., 2017)
Trastuzumab, Lapatinib	Breast cancer	SKBr-3	ErbB2↓; ROS↑	Produce higher cytotoxicity with substantially lower doses of celastrol	(Raja et al., 2011)
EGFR-TKIs	Lung cancer	A549 and H1975	EGFR, STAT3, pAKT, and p-ERK↓	Suppress invasion	(Wang et al., 2018)
SAHA	Lung cancer; Ovarian cancer	95-D and SK-OV-3	E-cadherin↑; caspase-3 and cleaved PARP↑	Enhance anticancer efficacy; celastrol and SAHA are reciprocal sensitization	(Zheng et al., 2014)
Sorafenib	Liver cancer	HepG2 and Hepa1-6	VEGF and P-AKT↑	Enhance the growth inhibition and apoptosis induction; celastrol enhanced the antitumor activity of sorafenib in HCC tumor cells by suppressing the AKT pathway and VEGF autocrine system	(Zhang et al., 2019)
PHA-665752	Liver cancer	BEL-7402 and Huh7	G2/M arrest↑; caspase3/7↑	Enhance the growth inhibition effect	(Jiang et al., 2013)
Apatinib	Liver cancer	Hep3B	p-Akt and p-ERK↓; Caspase-3 and Bax↑	Inhibit the proliferation, migration and invasion ability and promote the apoptosis	(Li, Fan, et al., 2018)
ABT-737	Liver cancer	Bel-7402 and HepG2	Caspase-3 and PARP, bax ↑; Bcl-2 and Bcl-xL, Mcl-1↓; bim and PUMA↑; Hsp90↓; ATF4, phosphorylation of eIF2a↑	Upregulate Noxa by ER stress	(Zhu et al., 2012)
17-AAG	GBM	U251N and U343	Polyubiquitinated aggregates↑; p62 accumulation↑	Sensitize human glioblastoma to celastrol treatment	(Boridy et al., 2014)
Sulfasalazine	Glioma	SNB-19	G2/M arrest↑; EGFR↓	Be effective both as an anticancer drug and as an agent for sensitizing gliomas to celastrol	(Pham et al., 2010)
5-Fluorouracil, salinomycin, 1400 W, and L-NIO	Colorectal cancer	HT-29 and HCT116	IL-1b, MMP-9, PDGF, Serpin E1, and TIMP-4↓	Enhance the efficacy of other chemotherapeutic drugs, including 5-FU, salinomycin, 1400 W, and L-NIO, in inhibiting CRC cell proliferation	(Gao et al., 2019)
Paclitaxel	ATC	8505C and SW1736	Phospho-ERK1/2, phospho-JNK, bip, and cox 2↑ ROS↑; hsp90, hsp70, bax, DR5, cleaved caspase-3, and cleaved PARP↑; ErbB2, Raf-1, and Bcl2↓	Celastrol synergized with paclitaxel in induction of cytotoxicity	(Kim et al., 2017)
Carboplatin	PHGG	VUMC-HGG-14	Ki-67↓; FANCD2↓	Degradation of FANCD2 via celastrol treatment sensitizes HGGs to carboplatin-mediated DNA damage	(Metselaar et al., 2019)
Cisplatin	OS	U-2OS	Caspase-3, cytochrome c(Cyt-c) and bax ↑; caspase-9, PARP, Bcl-2↓; GRP78↓, CHOP↑	Induce apoptosis via the mitochondrial and endoplasmic reticulum pathways	(Wang, Yu, et al., 2019)
Bortezomib	Multiple myeloma	U266	Caspase-3↑; NF-kB, CXCR4 and MMP-9↓; IL-6 and TNF-α↓	Reduce cell proliferation and enhance apoptosis; inhibit invasion and migration	(Shanmugam et al., 2018)
Temozolomide	Melanoma	SK-MEL-173	Ubiquitinated proteins↑; IκB phosphorylation↓; JNK phosphorylation↑	Celastrol may be effective in sensitizing resistant melanoma cells to the effects of temozolomide	(Chen et al., 2009)
Daunorubicin	Leukemia	K-562 and Jurkat T	Caspase 3↑; PARP cleavage↑; Bcr-Abl↓	Apoptosis induction; sensitize the effect of chemotherapy in human leukemia cells	(Davenport et al., 2010)
TNF-α	Breast cancer	MDA-MB-231	Caspase-3 and Caspase-9↑; NF-κB, XIAP↓	Sensitize breast cancer cells MDA-MB-231 to celastrol	(Lu et al., 2014)
TRAIL	Lung cancer	A549	Caspase 3 and 8↑; LC3-II and p62↑; ROS↑; ΔΨm↓	Celastrol-mediated autophagy flux inhibition sensitized TRAIL-initiated apoptosis via regulation of ROS and ΔΨm	(Nazim, Yin, and Park 2019)
TRAIL	GBM	U87-MG	Activation of caspase-8, caspase-3, and PARP↑; DR5↑	Celastrol sensitized glioma cells to TRAIL via the death receptor pathway; DR5 plays an important role in the effects of this cotreatment	(Cha et al., 2019)
TRAIL/APO-2L	Colon cancer; ovarian carcinoma	SW620 and OVCAR8	DR4 and DR5↑; caspase-3, PARP, XIAP↑	Enhance mRNA and protein expression of DR4 and DR5 play prominent roles in the sensitization of celastrol to TRAIL/Apo-2l-induced apoptosis, in a p38 MAPK-independent manner	(Zhu et al., 2010b)
TRAIL/APO-2L	Ovary cancer; colon cancer; Lung cancer	OVCAR-8, SW620 and 95-D	PARP, pro-caspase-3, caspase-9↑	Enhance anticancer activities by the prompt onset of caspase mediated apoptosis	(Zhu et al., 2010a)
Triptolide	Lung cancer	H1299 and H157	G2/M phase↑, G0/G1 phase↓, Cdk1, Cyclin B and p21↑, Cdk2/4/6, Cyclin D/E, pRb, Rb and p27↓; generated cleaved PARP and Caspase-3, bax and bcl-XS/l↑, Bcl-2, Mcl-1, survivin and XIAP↓; ROS levels↑; HSP90 client proteins including survivin, AKT, EGFR↓	Induce G2/M cell cycle arrest; induce cancer cell apoptosis; ROS is critical for the synergistic anticancer effects	(Jiang et al., 2015)
Ellagic acid	Lung cancer	HOP62 and H1975	LC3-II↑; CIP2A↓	Enhance autophagy and down-regulate CIP2A	(Duan et al., 2019)
Betulinic acid	Marek’s disease	MSB-1 and BT-474	p65 and Meq↓; IκB↑	Anti-proliferation and apoptosis; inhibition of NF-κB transcriptional activity; target antiapoptotic gene Meq	(An, Li, and Zhang 2015)
Gambogic acid	OSCC	Tca8113, TSCC and NT	NF-kappa B↓	Inhibit the proliferation and induce the apoptosis	(He et al., 2009)
Ionizing radiation	Lung cancer	NCI-H460	Hsp90: EGFR, ErbB2 and survivin ↓; p53, phosphorylating Ser15 and Ser20↑	Enhancement of radiation sensitivity	(Lee et al., 2011)
Ionizing radiation	Prostate cancer	PC-3	γH2AX levels↑; Mcl-1 and PARP↑	Sensitize PC-3 cells to radiation both *in vitro* and *in vivo* by impairing DNA damage processing and augmenting apoptosis	(Dai et al., 2009)
Hsp70 siRNA	Glioblastoma	U251N	Silence inducible Hsp70	Reduce cell viability, and enhance antiproliferative effects	(Matokanovic et al., 2013)
miR-101 mimics	Prostate cancer	LNCaP	p62↓; AR expression↓; miR-101↓	Autophagy inhibition by miR-101 mimic was found to enhance the cytotoxic effect of celastrol in prostate cancer cells	(Guo et al., 2015)
miR-33a-5p	Lung cancer	A549 and LTEP-a-2	mTOR, p-p70S6K and p-4EBP1↓	Enhance the sensitivity of lung adenocarcinoma cells to celastrol	(Li, Sun, et al., 2018)
Immunotherapy	OS	HOS and U2OS	DR4/5↑; cancer cell lysis by γδ T cells↑	In combination with immunotherapy approaches employing adoptive γδ T cell transfer	(Li et al., 2016)

TNF-α, tumor necrosis factor α; TRAIL, tumor necrosis factorrelated apoptosis-induced ligand; EGFR-TKI, epidermal growth factor receptor tyrosine kinase inhibitor; SAHA, suberoylanilide hydroxamic acid; 17-AAG, 17-N-Allylamino-17-demethoxygeldanamycin; ATC, anaplastic thyroid carcinoma; pHGG, pediatric high-grade gliomas; OS, osteosarcoma; OSCC, oral squamous cell carcinoma; GBM, glioblastoma multiforme; DR5, death receptor 5; PARP, poly(adenosine triphosphate-ribose) polymerase; ROS, reactive oxygen species; TRAIL/APO-2L, tumor necrosis factor (TNF) α–related apoptosis-inducing ligand; ΔΨm, mitochondrial membrane potential.

**TABLE 2:** Summary of the antitumor effects of celastrol and chemotherapeutic drugs *in vivo*.

Treatment	Type of cancer	Animal model	Findings	Ref
Carboplatin	PHGG	Female athymic nude mice were stereotactically injected VUMC-HGG-14 cells (50 × 104 cells in 5 μL) into the striatum	Prolong survival of pHGG-bearing mice; combination therapy using celastrol and carboplatin might serve as a clinically relevant strategy for the treatment of pHGG	(Metselaar et al., 2019)
Bortezomib	Multiplemyeloma	Male athymic balb/c nude mice were implanted with 2 × 106 cells with human MM U266 cell lines subcutaneously	Augmented bortezomib induced inhibition of tumor growth; No any obvious side effects	(Shanmugam et al., 2018)
EGFR-TKIs	Lung cancer	BALB/c nude mice were subcutaneously injected with 2 × 106 H1975 lung carcinoma cells	Inhibit tumor growth	(Wang et al., 2018)
SAHA	Lung cancer	Human lung cancer 95-D xenografts were established by subcutaneously inoculating 5 × 106 cells into nude mice	Tumor growth inhibition without increased toxicity	(Zheng et al., 2014)
Sorafenib	Liver cancer	The C57bl/6 mice were injected subcutaneously with Hepa1-6 single-cell suspension cells (2 × 107/ml) into the right ﬂank	Enhance the antitumor activity and reduce the dosage of sorafenib	(Zhang et al., 2019)
PHA-665752	Liver cancer	Male nude mice were inoculated subcutaneously with human liver cancer cell lines Huh7	The combination of celastrol and PHA could effectively inhibit c-met-deficient hepatocellular carcinoma cells growth, migration and apoptosis	(Jiang et al., 2013)
Trastuzumab, Lapatinib	Breast cancer	Female NODSCID mice received sub-cutaneous 17β-estradiol pellet (0.72 mg/day), 2 weeks prior to injection of 5 x 106 BT-474 cells resuspended in 4% Matrigel	Retard the rate of growth of ErbB2- overexpressing human breast cancer cells in a mouse xenograft model with only minor systemic toxicity	(Raja et al., 2011)
X66	Breast cancer	Female Balb/cA-nude mice were implanted subcutaneously with BT-474 cells in the right ﬂank	Enhance anti-tumor activity, with no additional toxicity	(Zhao et al., 2016)
TRAIL/APO-2L	Lung cancer	Human lung cancer 95-D xenografts were established by 5 × 106 cells subcutaneously inoculated in nude mice	Increase the *in vivo* antitumor capacities without increasing the toxicities caused by the celastrol	(Zhu et al., 2010a)
TRAIL/APO-2L	Colon cancer	Human colon cancer SW620 xenografts were established by 5 × 106 cells subcutaneously inoculated in nude mice	Inhibit tumor growth	(Zhu et al., 2010b)
Triptolide	Lung cancer	Balb/c nude mice were injected subcutaneously with H1299 or H157 cells (3 × 106 in 100 μL of medium) under the shoulder	Inhibit the growth of tumors without obvious toxicity	(Jiang et al., 2015)
Ellagic acid	Lung cancer	Female BALB/C nude mice were injected HOP62 cells (1 × 106) subcutaneously into the right rear flank	Inhibitory effects on tumor growth, without a reduction in body weight	(Duan et al., 2019)
miR-33a-5p	Lung cancer	BALB/c-nu/nu male mice were injected subcutaneously with LTEP-a-2 cells into the right or left ﬂanks	miR-33a-5p inhibited the proliferation of lung adenocarcinoma cells, enhanced the antitumor effect of celastrol, and improved sensitivity to celastrol by targeting mTOR	(Li Y. J. et al., 2018)
Ionizing radiation	Prostate cancer	Female athymic NCr-nu/nu mice were inoculated subcutaneously on both sides of the lower back above the tail with 3 × 106 cells/0.2 ml of PC-3 cells	Inhibit PC-3 tumor growth without obvious systemic toxicity	(Dai et al., 2009)
Ionizing radiation	Lung cancer	Nude mice were injected with 5 × 10^7^ A549 cells in the back subcutaneously	Radiosensitizing agent celastrol has therapeutic effects when combined with ionizing radiation	(Jun et al., 2017)

EGFR-TKI, epidermal growth factor receptor tyrosine kinase inhibitor; SAHA, suberoylanilide hydroxamic acid; TRAIL/APO-2L, tumor necrosis factor (TNF) α–related apoptosis-inducing ligand; X66, 4-(2-((1H-indol-3-yl)methylene)hydrazinyl)-N-(4-bromophenyl)-6-(3,5- dimethyl-1H -pyrazol-1-yl)-1,3,5-triazin-2-amine.

#### Combination With Chemotherapeutic Agents

##### Improvement of the Therapeutic Effect

The combination of chemotherapy drugs can produce an increased or synergistic effect and improve the therapeutic effect. It is the main mode of clinical tumor treatment (Ashton, 2015). Yan et al. (2017) combination celastrol and lapatinib produced strong synergy in growth inhibition and apoptosis in comparison to single-agent treatment in HER2/neu-overexpressing MDA-MB-453 cells. Recently, the study (Li H. et al., 2018) aimed to investigate the effects of apatinib and celastrol on the proliferation, invasion and apoptosis of human hepatoma Hep3B cells. Finding it can produce a synergistic effect by downregulating the expression of p-Akt and p-ERK, and upregulating the expression of Caspase-3 and Bax. Celastrol was also described to enhance the anti-liver cancer activity of sorafenib (Zhang R. et al., 2019). Celastrol enhanced the antitumor activity of sorafenib in hepatocellular carcinoma (HCC) tumor cells by suppressing the AKT pathway and VEGF autocrine system, and enhanced the growth inhibition and apoptosis induction of cancer cells by sorafenib both *in vitro* and *in vivo* and reduced the dosage of sorafenib needed. Additionally, celastrol combined with epidermal growth factor receptor tyrosine kinase inhibitors significantly suppressed cell invasion of lung cancer cells with a T790M mutation by suppressing the EGFR pathway. And combined therapy can also inhibit tumor growth *in vivo* (Wang et al., 2018). Researchers investigated that combining ABT-737 and celastrol synergistically suppressed HCC cell proliferation, and induced apoptosis which was accompanied with the activation of caspase cascade and release of cytochrome c from mitochondria. Further study revealed that the enhanced Noxa caused by celastrol was the key factor for the synergy, since small interfering RNA-mediated knockdown of Noxa expression in HCC cells resulted in decreased apoptosis and attenuated anti-proliferative effects of the combination. In addition, it unraveled that, upon celastrol exposure, the activation of endoplasmic reticulum stress, specifically, the eIF2a-ATF4 pathway played indispensable roles in the activation of Noxa, which was validated by the observation that depletion of ATF4 significantly abrogated the Noxa elevation by celastrol (Zhu et al., 2012).

The free radical nitric oxide (NO) is known to play a critical role in colorectal cancer growth by promoting tumor angiogenesis. Study indicated that the antiproliferation activity of celastrol was associated with the inhibition of iNOS and eNOS in colorectal cancer cells (Gao et al., 2019). Therefore, treatment with celastrol inhibited colorectal cancer cell growth and migration, and was associated with suppression of the expression of key genes [TYMP, CDH5, THBS2, LEP, MMP9, and tumor necrosis factor α (TNF)] and proteins (IL-1b, MMP-9, PDGF, Serpin E1, and TIMP-4) involved in the angiogenesis pathway. In addition, combinational use of celastrol with 5-fluorouracil, salinomycin, 1400W, and L-NIO showed enhanced inhibition of colorectal cancer cell proliferation and migration.

Also, studies have shown that celastrol at high concentration (>1.0 μM) induced G2/M arrest and apoptosis with the activation of caspase3/7 in Huh7 cells whereas at low concentration (<1.0 μM) had no obvious effects. Low concentration celastrol presented significant combined effects with PHA on Huh7 cells and Huh7 xenografts in terms of growth inhibition, migration inhibition and apoptosis induction (Jiang et al., 2013).

##### Reduction of Related Adverse Effects of Celastrol

Lowering the dosage used of celastrol by combining it with chemotherapeutic agents effectively reduces its related adverse effects. During the study carried out by Raja et al. (2011) the researchers observed that the Michael Acceptor functionality in celastrol is important for its ability to destabilize ErbB2 and exert its bioactivity against ErbB2-overexpressing breast cancer cells. Because celastrol not only induced the expected ubiquitinylation and degradation of ErbB2 and other HSP90 client proteins, but it also increased the levels of ROS. Therefore, celastrol strongly synergized with ErbB2-targeted therapeutics trastuzumab and lapatinib, producing higher level of killing of ErbB2-overexperssing breast cancer cell lines SKBr-3 with substantially lower doses of celastrol. Meanwhile, the investigators examined the efficacy of celastrol against ErbB2-overexpressing BT-474 cell line in a NOD-SCID xenograft model. Celastrol signifcantly retarded the rate of growth of ErbB2-overexpressing human breast cancer cells *in vivo* with only minor systemic toxicity. Similarly, a study utilizing BT-474 xenograft model determined that combining X66 with celastrol could deplete client protein and inhibit tumor growth, and lead to enhanced activity. No additional toxicity was observed in the co-treatment group as assessed by treatment-related mortality and body weight change (Zhao et al., 2016). As reported, celastrol and cisplatin inhibited the growth of U-2OS cells in a dose-dependent manner. And it induced apoptosis in U-2OS cells via the mitochondrial and endoplasmic reticulum pathways (Wang Q. et al., 2019). Celastrol significantly reduced cell proliferation and enhanced apoptosis when used in combination with bortezomib and upregulated caspase-3 in these cells (Shanmugam et al., 2018). Celastrol also augmented bortezomib induced inhibition of tumor growth in statistically significant manner. Moreover, it did not notice any obvious side effects from the administration of celastrol. Hence, published data suggest that the combination therapy of celastrol can play the role of increasing therapeutic effect and reducing toxicity.

##### Overcoming Multidrug Resistance

In addition, due to the different mechanism of action of chemotherapy drugs, it could prevent the occurrence of multidrug resistance (MDR). In a study (Pham et al., 2010), it was shown that sulfasalazine, a Food and Drug Administration-approved drug, may be effective both as an anticancer drug and as an agent for sensitizing gliomas to celastrol. Similarly, a study emphasized that targeting proteotoxic stress responses by inhibiting HSP90 with 17-AAG sensitized human glioblastoma to celastrol treatment, thereby serving as a novel synergism to overcome MDR (Boridy et al., 2014). Research reported that E-cadherin expression, as a classic marker that is used to define either epithelial or mesenchymal characteristics, was reduced in celastrol-resistant SK-OV-3 cells (Hayashi et al., 2010). In another study showed that the synergistic anticancer effects of celastrol and suberoylanilide hydroxamic acid (SAHA) due to their reciprocal sensitization, which was simultaneously regulated by NF-κB and E-cadherin. (Zheng et al., 2014). So SAHA increased the expression of E-cadherin which could significantly augmented the ability of celastrol monotherapy to induce apoptosis. Due to the sensitivity of celastrol to SK-OV-3 cells is improved, it can indirectly play the role of anti-MDR. Moreover, the enhanced anticancer efficacy of celastrol combined with SAHA was validated in a human lung cancer 95-D xenograft model without increased toxicity. The findings not only open new opportunities for the clinical development of SAHA but should also motivate the clinical investigation of celastrol, which has been hampered by its toxicity.

On the other hand, celastrol, as a blood-brain barrier-penetrable compound, could degrade FANCD2, to sensitize glioma cells to the archetypical DNA-crosslinking agent carboplatin *in vitro* in seven patient-derived pediatric high-grade gliomas (pHGG) models (Metselaar et al., 2019). Therefore, combination therapy using celastrol and carboplatin might serve as a clinically relevant strategy for the treatment of pHGG.

#### Combination With Tumor Necrosis Factor Superfamily

Tumor necrosis factor superfamily can specifically induce programmed apoptosis of tumor cells, but it is not toxic to normal cells (Aggarwal et al., 2012). Therefore, it has become a hot research field of anti-tumor in recent years. In a study, it has been showed that TNF-α could sensitize breast cancer cells MDA-MB-231 to celastrol through inhibiting the activation of NF-κB signaling, leading to XIAP inhibition with subsequent upregulation of caspase-3 and caspase-9 activities (Lu et al., 2014). Thus, it indicated when combined with the natural proteasome inhibitors, celastrol, the anti-cancer activities of TNF-α can enhance. In addition, Nazim et al, (2019) have shown that treatment with celastrol caused an increase in microtubule-associated proteins 1A/1B light chain 3B-II and p62 levels, whereas co-treatment of celastrol and tumor necrosis factorrelated apoptosis-induced ligand (TRAIL) increased active caspase-3 and caspase-8 levels compared with the control, confirming inhibited autophagy flux. Therefore, the combined use of TRAIL with celastrol may serve as a safe and adequate therapeutic technique for the treatment of TRAIL-resistant lung cancer. Other studies (Cha et al., 2019) demonstrated that celastrol sensitized glioma cells to TRAIL via the death receptor pathway and that DR5 played an important role in the effects of this co-treatment. It indicates that this co-treatment is a promising tumor-killing therapeutic strategy with high efficacy and low toxicity. Zhu et al. (2010a), Zhu et al. (2010b) reported combining TRAIL/APO-2L and celastrol could produce synergistic anticancer capabilities. On the one hand, the enhanced mRNA and protein expression of DR4 and DR5 play prominent roles in the sensitization of celastrol to TRAIL/Apo-2L-induced apoptosis, in a p38 MAPK-independent manner. On the other hand, these enhanced anticancer activities were accompanied by the prompt onset of caspase mediated apoptosis. It opens new opportunities to enhance the effectiveness of future treatment regimens using TRAIL/APO-2L.

#### Combination With Active Ingredients of Traditional Chinese Medicine

He and co-workers found that the combination of gambogic acid and celastrol has a synergistic antitumor effect for treating oral squamous cell carcinoma. The minimal cytotoxic dose of celastrol was able to effectively suppress the gambogic acid-induced NF-kappa B pathway activation (He et al., 2009). Moreover, active components from the same herb can synergize with other compounds isolated from the same herb (Zhou et al., 2016). For example, combination treatment of triptolide and celastrol synergistically inhibits cell growth, induces cell cycle arrest at G2/M phase and apoptosis, and increases intracellular ROS accumulation in many types of cancer cells, including H1299 and H157 lung cancer cells (Jiang et al., 2015).

It has also been determined that 10–50 μM ellagic acid significantly enhanced the effects of celastrol (at relatively low concentrations) on lung cancer cells by inducing autophagy. It also found that combined treatment resulted in significant inhibitory effects on tumor growth compared with either monotherapy, without a reduction in body weight. These data indicate that the combination of ellagic acid and celastrol exerts synergistic anti-lung cancer effects both *in vitro* and *in vivo* (Duan et al., 2019). Another study (An et al., 2015) suggested that the combination of betulinic acid and celastrol at lower concentration may produce a synergistic inhibitive effect on MSB-1 cells. Besides, betulinic acid and celastrol of the above concentrations are nontoxic to non-tumorous chicken embryo fibroblasts and selective to MSB-1 cells. Thus warrants further investigation for its potential clinical applications.

#### Combination With Ionizing Radiation

It has also been found that celastrol may be considered an effective radiosensitizer acting as an inhibitor of Hsp90 and a p53 activator. The two activities could be applicable to a broad range of cancer cells with either wild-type or mutant p53 because either activity could be effective for the enhancement of radiation cell killing (Lee et al., 2011). The study showed that the novel radiosensitizing agent celastrol had therapeutic effects when combined with ionizing radiation (IR) *in vitro* and *in vivo* (Jun et al., 2017). Besides, diffusion-weighted magnetic resonance imaging was a useful noninvasive tool to monitor the effects of celastrol by assessing cellularity changes and sequential therapeutic responses. Furthermore, when combined with IR, celastrol significantly prolonged the presence of IR-induced γH2AX and increased IR-induced apoptosis (Dai et al., 2009). Celastrol, combined with fractionated radiation, significantly inhibited PC-3 tumor growth *in vivo* without obvious systemic toxicity. Therefore, the research outputs celastrol sensitized PC-3 cells to radiation both *in vitro* and *in vivo* by impairing DNA damage processing and augmenting apoptosis.

#### Combination With Nucleic Acid

The miRNA is a short endogenous noncoding RNA and responsible for the modulation of cell migration, proliferation, programmed cell death, and cell differentiation by directly binding to target gene mRNA and further leading to mRNA degradation and translational inhibition via imperfect base pairing to the 3′-UTR (Huntzinger and Izaurralde, 2011). Besides, miRNAs can also function as oncogenes or tumor suppressor genes in carcinogenesis, cancer progression, and metastasis (Huang et al., 2017). Guo et al. (2015) found that autophagy inhibition by miR-101 mimic could enhance the cytotoxic effect of celastrol in prostate cancer cells. Similarly, findings also suggested that miR-33a-5p could inhibit the proliferation of lung adenocarcinoma cells, enhance the antitumor effect of celastrol, and improve sensitivity to celastrol by targeting mTOR in lung adenocarcinoma *in vitro* and *in vivo* (Li Y. J. et al., 2018). In addition to this, inducers of heat shock protein 70 (Hsp70) commonly promote cancer cell viability whereas inhibitors of Hsp90 reduce it. Therefore, in order to silence inducible Hsp70 and to promote celastrol-induced tumor cell death, Matokanovic and co-colleagues prepared Hsp70 siRNA loaded chitosan-TPP carriers and it is found that silencing of Hsp70 by chitosan-TPP-Hsp70 siRNA treatment significantly reduced cell viability, and enhanced antiproliferative effects of celastrol in glioblastoma cells (Matokanovic et al., 2013). Thus combination of nucleic acid with celastrol may represent a promising therapeutic approach for treating cancers.

### Structural Derivatives of Celastrol

**FIGURE 3:**
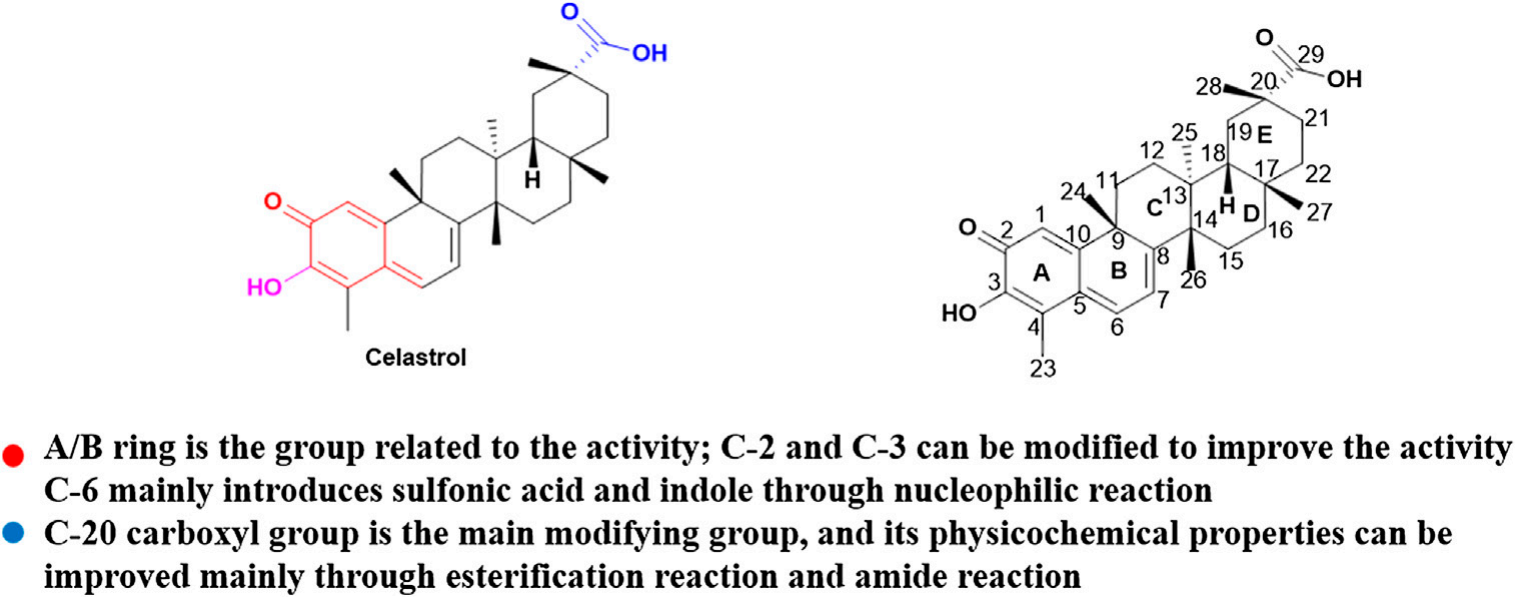
The structure of celastrol and its some important properties.

Celastrol has poor water-solubility, short half-life, narrow therapeutic window and low bioavailability, which seriously affect its clinical application. With the development of synthetic technology, the structure of celastrol was modified by chemical means and the derivatives with biological activity were synthesized. At present, it is mainly by modifying the A/B ring and position C-20 (Figure 3) to improve the properties of the drug and enhance the anti-tumor activity.

#### Increase Solubility to Enhance Antitumor Effects

**FIGURE 4:**
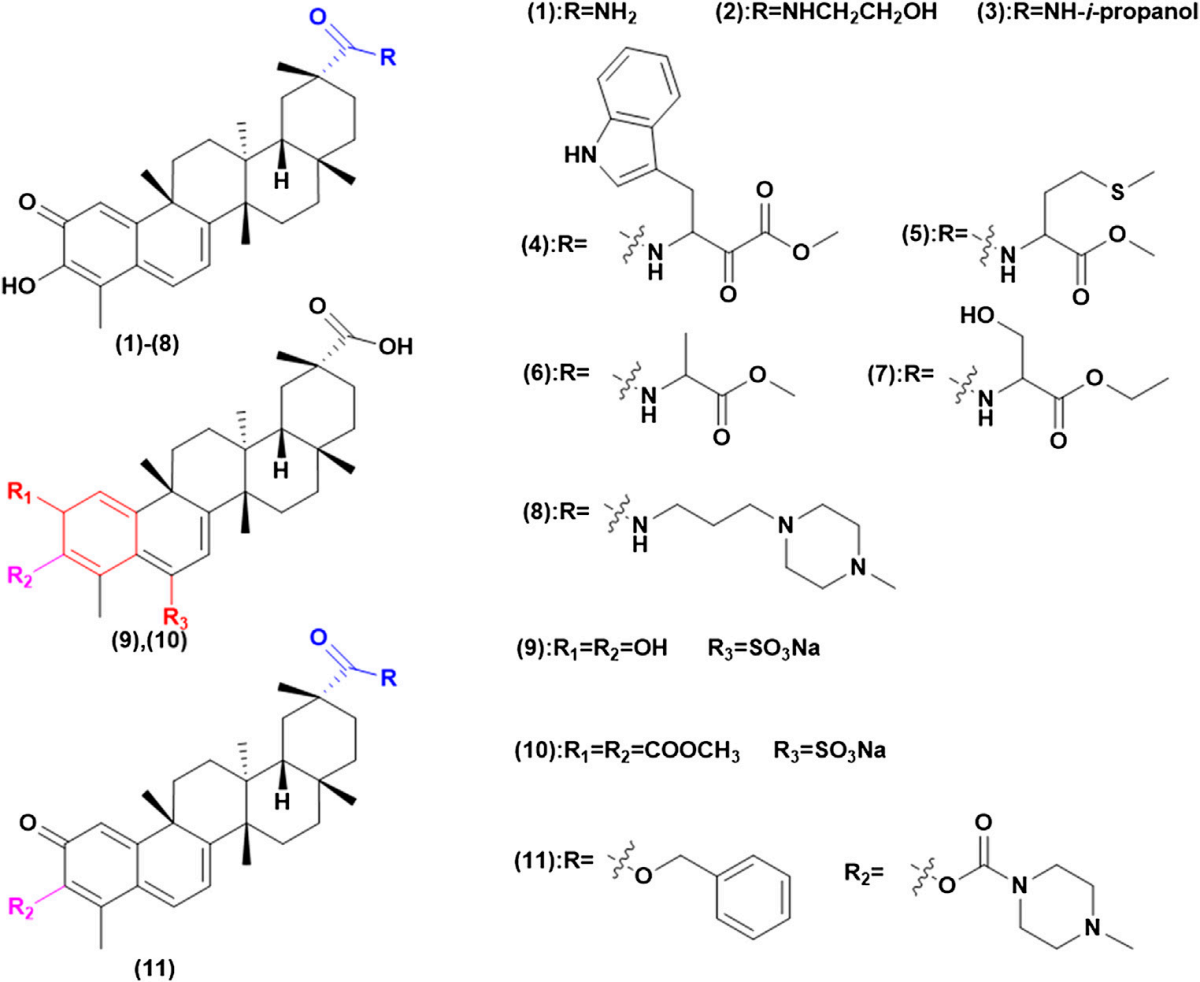
Increase solubility to enhance antitumor effects.

Celastrol has poor water-solubility, which greatly limits its bioavailability. Therefore, enhancing the solubility of celastrol is conducive to improving the antitumor effects. Generally speaking, the solubility of compound is directly related to the number of polar groups. By introducing hydrophilic groups such as amino, hydroxyl, amide, and sulfonic acid, the solubility is improved and the efficacy is studied (Cai et al., 2016) (Figure 4). Klaić et al. (2012) converted carboxylic acid functional groups into amides, of which compound (1) can enhance the induction of heat shock response of the matrix, due to the hydrophilic groups increasing solubility and permeability. Tang and his teamworkers (Tang et al., 2015) synthesized a series of celastrol derivatives through amide reaction at the C-20 site, these compounds are effective against SGC-7901, SMMC-7721, MGC-803 and HepG 2 cell lines proliferation. They found that derivatives (2) and (3) contained hydrophilic groups to improve solubility and then had high antitumor effects. Their inhibitory IC_50_ on telomerase was 0.11 and 0.34 mM, which increased the anti-proliferation activity of HepG 2 cells by 6.6 times and 14.3 times. In this study, the tumor target telomerase was associated with celastrol for the first time, and celastrol derivatives potentially inhibiting telomerase were synthesized. Other researchers have modified amino acids, amines, and piperazine on C-20 to synthesize a series of celastrol derivatives with amide bonds, which has better solubility and penetration. Such as amino acids modified compound (4) has higher anticancer activity in AGS cell line with its IC_50_ value concentration of 0.44 μM, while the concentration of compound (5) *in vivo* was higher than that of celastrol. Besides, compounds (6) and (7) have a strong antiproliferative activity of Hela and A549 cell lines, which are 5 and 10 times that of celastrol (Zhang H.-J. et al., 2018; Pang et al., 2018; Wang G. et al., 2019). On the other hand, the water solubility of compound (8) is 35 times that of celastrol and its tumor suppressive activity is higher than celastrol against the PANC-1 cell line and the human lung cancer (paclitaxel-resistant) A549 cell line (Jiang et al., 2016).

In addition, Zeng and Tang synthesized compounds (9) and (10), through the introduction of C-6 sulfonamide salt, which greatly increases its solubility. The results showed it could significantly inhibit tumor growth at low concentrations (Zeng et al., 2012; Tang et al., 2014). Meanwhile Shan and his team (Shan et al., 2017) treated amino acid methyl hydrochloric acid on the C-3 and C-20 position, in which compound (11) was introduced by piperazine to expand the electron cloud density of ring A and reduce the toxicity during *in vivo* experiment by intragastric administration.

#### Increase Stability to Enhance Antitumor Effects

**FIGURE 5:**
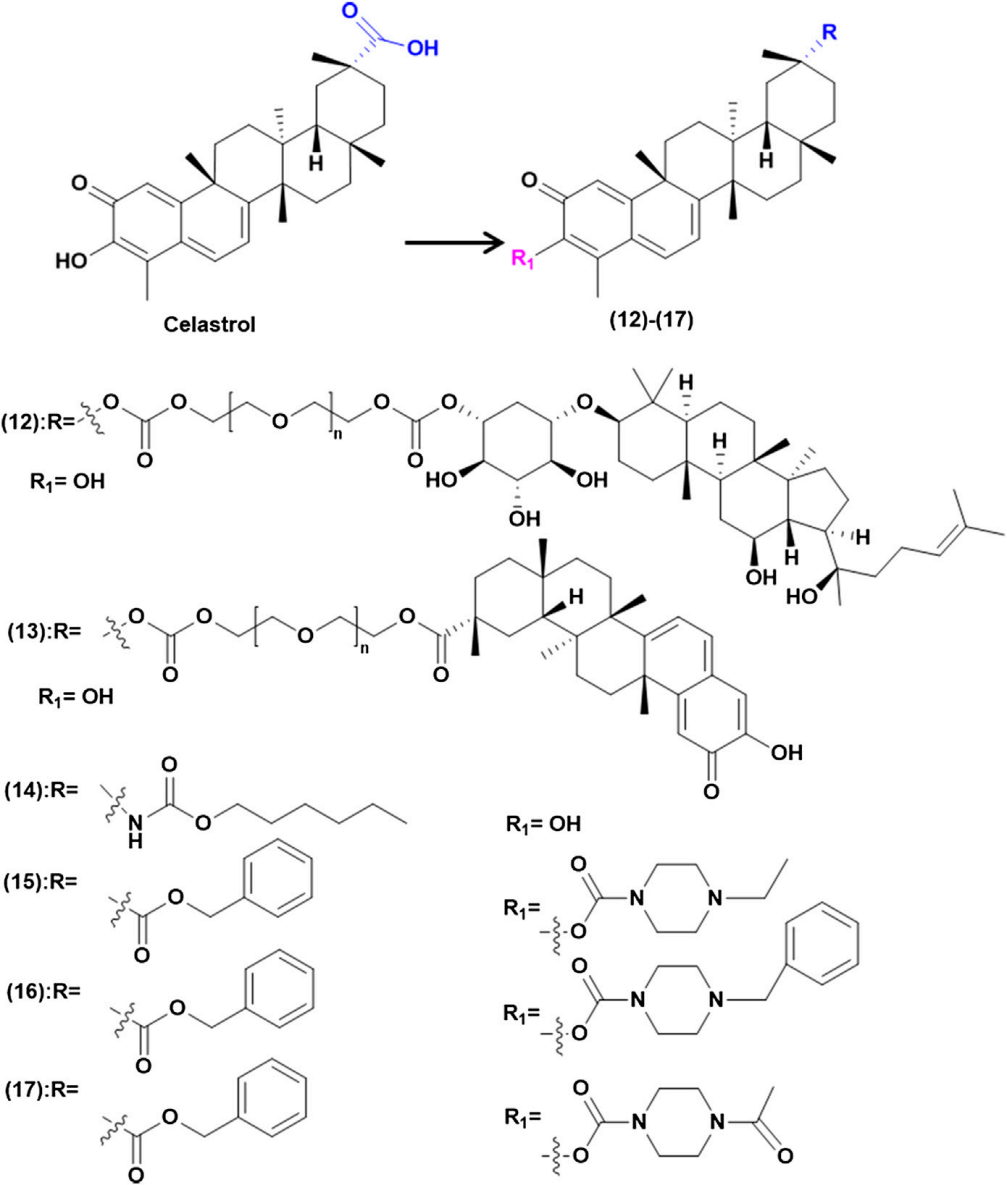
Increase stability to enhance antitumor effects.

Celastrol is easy to polymerize and unstable in acidic or alkaline environment, in which ring A is prone to ring opening and rearrangement (Shi 2015). Researchers modified the structure of celastrol to improve its stability (Figure 5). It was found that PEG has the function of long circulation, and the stability can be improved by linking celastrol to PEG. Li and other colleagues (Li et al., 2017) reported that celastrol and ginsenoside Rh2 were connected through PEG to synthetic compound (12), which could form micelles with a half-life increasing 1.03–2.44 times. The A549 cell absorption rate increased by 5.8 times, which made it higher cell uptake, stronger induction of apoptosis and anti-proliferation activity compared with celastrol. In another study, Shan and colleagues (Shan et al., 2019) studied celastrol derivatives by combining carboxyl groups on C-20 with PEG covalent bonds, the compound (13) can form micelles in water, which greatly improve the stability of celastrol. At the same time, through the experiment of A549 xenograft nude mice, it was proved that compound (13) has higher activity and safety than celastrol. Carbamate chemistry is a key structural motif for many marketed drugs and precursors because of its stability and ability to permeate cell membranes (Ghosh and Brindisi, 2015). So Figueiredo and others (Figueiredo et al., 2017) synthesized a series of diacetic carbamate derivatives by C-20 modification with celastrol, among which compound (14) had high activity. Meanwhile Shan and his team (Shan et al., 2017) treated amino acid methyl hydrochloric acid on the C-3 and C-20 position, in which compound (15)–(18) showed higher inhibition rate and better safety than celastrol during *in vivo* experiment by intragastric administration.

#### Improve Selectivity to Enhance Antitumor Effects and Reduce Toxicity

**FIGURE 6:**
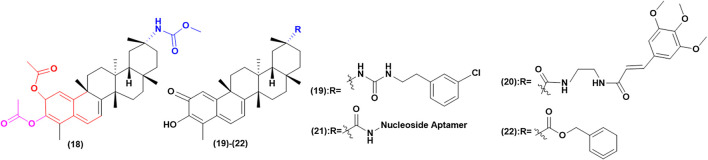
Improve selectivity to enhance antitumor effects and reduce toxicity.

Celastrol has the duality of “toxin-effect” and its active part is also the toxic part. By enhancing the selectivity of celastrol, the toxicity can be greatly reduced and the property of drug can be improved (Figure 6). Figueiredo’s team (Figueiredo et al., 2017) modified the C-20 position with urea and conducted *in vitro* experiments on SKOV-3 cells as well as on non-tumor BJ cells, shown that the activity of compound (19) in SKOV-3 cells is six times that in non-tumor BJ cells. The result indicated that the introduction of urea could greatly improve its selectivity and its activity is three times that of celastrol. They then modified C-20, C-2 and C-3 positions to synthesize celastrol derivatives of diacetate carbamate and A/B epoxy, compared with the parent compound, all diacetate showed higher cytotoxic activity to MIA PaCa-2. Among these compounds, carbamate derivative (20) has the highest activity and the lowest IC_50_. It has the activity of inhibiting the proliferation of various tumor cells. Besides, *in vitro* experiments showed that compound (20) had obvious selectivity between tumor cells and non-tumor human BJ cells. In addition, SKOV-3 cells were more sensitive to compound (20) than other tested cancer lines, which showed a 7-fold increase in tumor sensitivity to nontumor fibroblast cell lines. Liu (2017) innovatively linked the nucleoside aptamer to C-20 through celastrol to synthesize compound (21). Through *in vitro* cytotoxicity experiments on PANC-1 and normal human liver cell line, it was proved that compound (21) has higher anti-proliferative and growth activities, has less liver toxicity and high selectivity compared with celastrol. It was found that compound (22) were synthesized by linking with aromatic groups or cinnamamide, and *in vitro* experiments showed that its selective activity was increased (Li X. et al., 2019). Some scholars introduced aromatic substituted phenyl group to synthesize compound (23) and found that it has higher anticancer activity and lower cytotoxicity to normal cells (Wei et al., 2014; Shan et al., 2017).

#### Other Structural Modifications to Enhance Biological Activity

**FIGURE 7:**
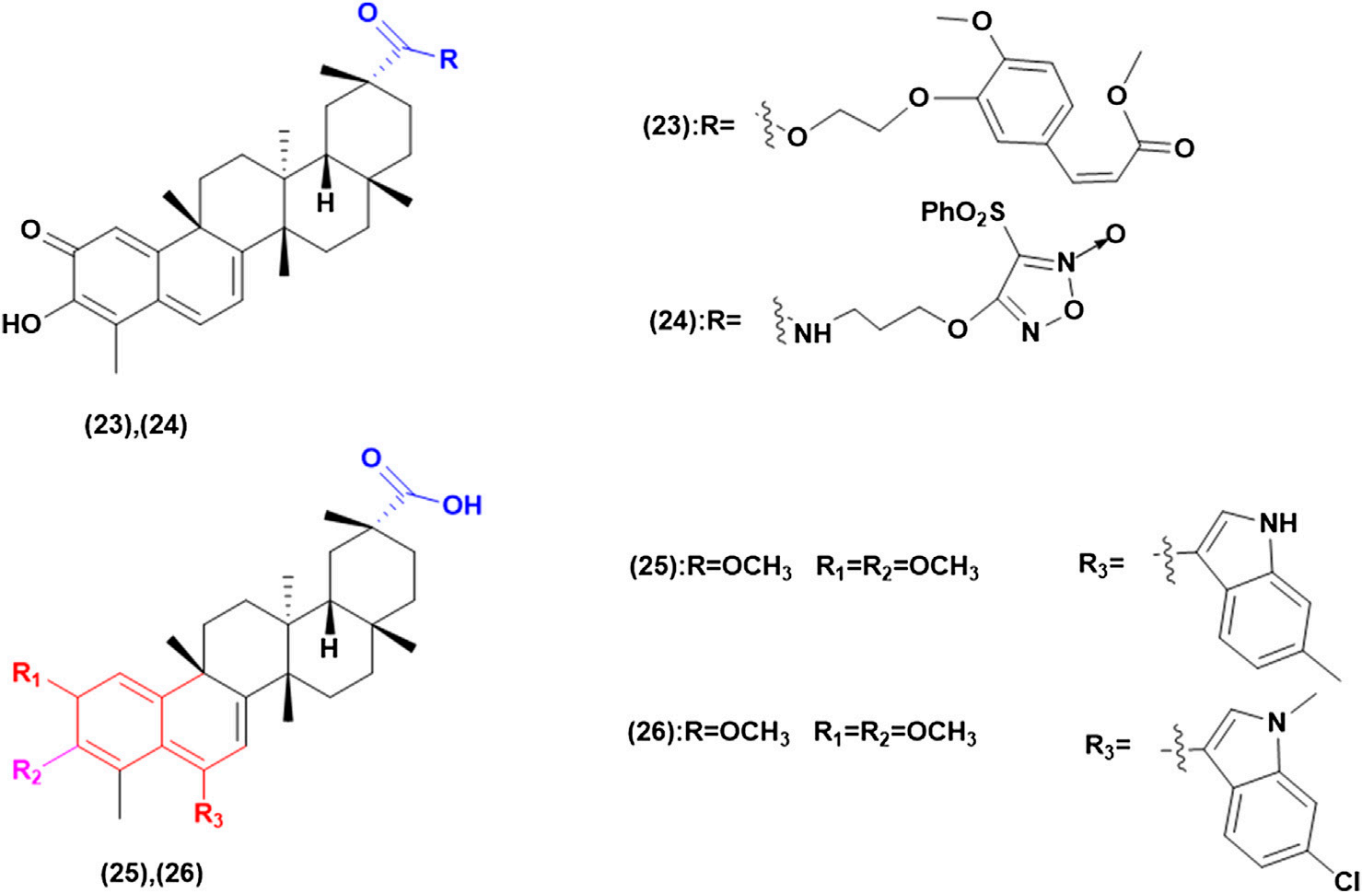
Other structural modifications to enhance biological activity.

In addition to the above, there are other structural modification derivatives that enhance biological activity (Figure 7). The inhibition of celastrol on protein HSp90 was found to have significant stereospecific specificity (Klaic et al., 2011), while modifications to C-20 and C-6 whose specific substituents can affect activity (Shan et al., 2017). Xu et al. (2019) adopted the natural product hybridization strategy, and the carboxyl group 29 was modified by methylferulic acid and its derivatives through different ligands. In particular, compound (24) had the ability to disturb the Hsp90-CDC37 complex stronger than celastrol, and its antitumor capacity was about 5 times that of celastrol.

(Li N. et al., 2018) used esterification and imitization at position C-20 to introduce furoxy NO donors into celastrol to synthesize celastrol/furoxy hybrids. Their antiproliferative to A549, HOS, MCF-7 and HepG2 were evaluated. The results showed it has higher biological activity. Moreover, it was found that anti-proliferative activity was positively correlated with the amount of NO released. In addition, the introduction of different types of NO donors has different effects on the structure of celastrol. In this study, compound (25) was screened out to show extremely strong activity in A549 cells (IC_50_ = 0.48 ± 0.06 M), greatly optimizing the activity of celastrol. Further mechanism studies showed that it could inhibit the activity of Hsp90 well and release high levels of NO together to induce apoptosis.

Additionally, some scholars have modified C-6 to improve the biological activity of celastrol. Such as, C-6 indole substituted derivatives, through cytotoxicity tests on human HCC Bel7402 and human glioblastoma cell line H4, of which compounds (26) and (27) have excellent anti-proliferative activity against Bel7402 cancer cells (IC_50_ = 0.02 and 0.01 μM). It is the first time to synthesize the derivative with C-C bond at C-6 site and anticancer activity *in vitro*. It is believed that Michael receptor is not necessary for its antitumor activity, which provides a prospect for the optimization of celastrol derivatives of this series (Tang et al., 2015).

### Nano/Micro-Systems Encapsulated Celastrol

**FIGURE 8:**
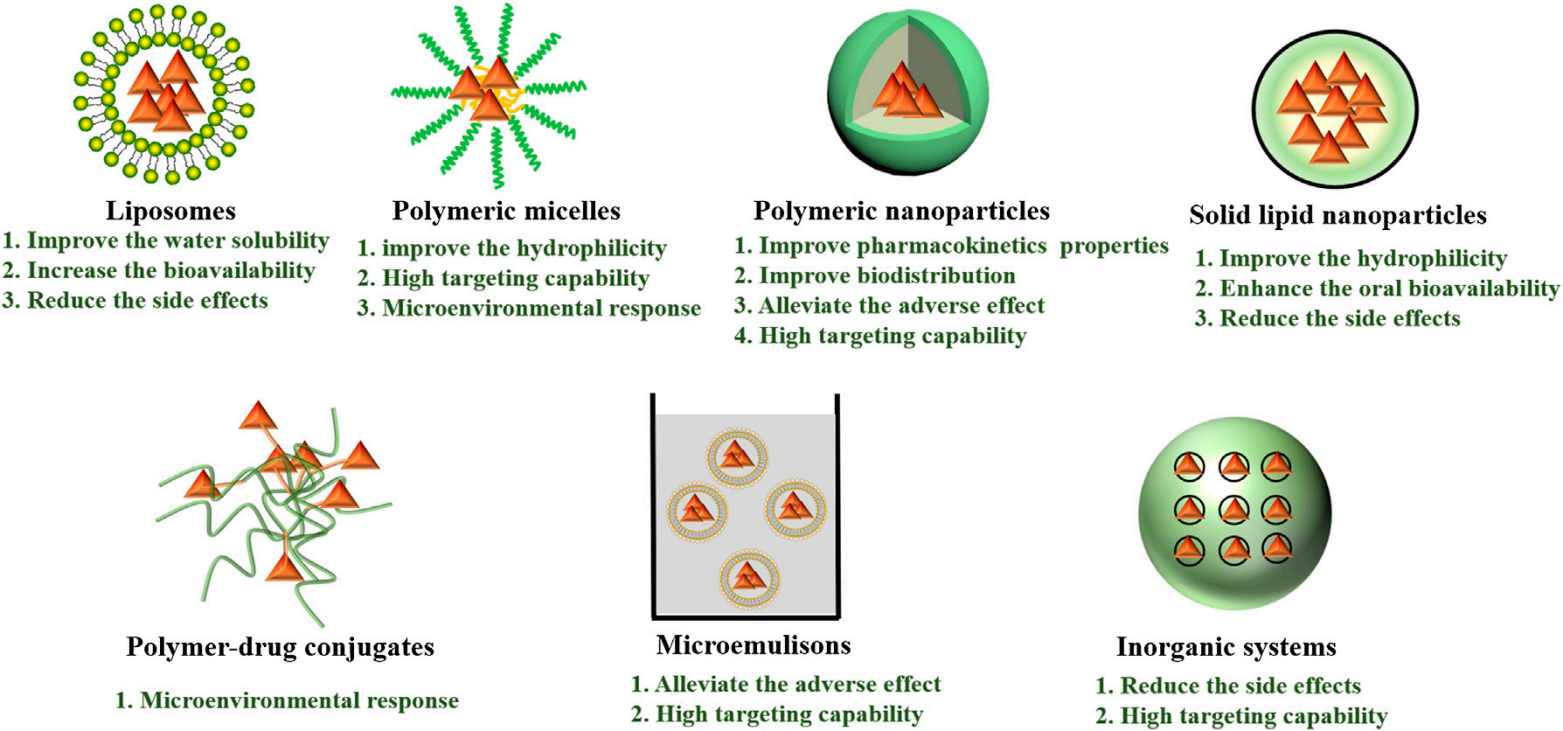
Different types of celastrol nano/micro-systems with their major advantages.

Nano/micro-science generated nano/micro-size vehicle-based drug delivery systems have greatly facilitated the precise delivery of drugs to target cells or tissues. As a great excitement advancement in the area of targeted delivery, it possesses various advantages, such as enhancing target ability to target cells or tissues, overcoming drug resistance by intracellular delivery, and realizing sustained and controlled release (Bernabeu et al., 2017; Zhou et al., 2017). What’s more, nano/micro-systems could also change the pharmacokinetics and toxicity profiles of parental drugs, enable drugs specific accumulation in the tumor tissue and release drugs at a synchronized rate, thereby maintaining synergistic drug ratios to achieve enhanced antitumor effects (Zhang, et al., 2017; Zhang J. et al., 2017; Xu and Liu 2019). Nowadays, different types of nano/micro-carriers have been employed to improve celastrol aqueous solubility, chemical stability, efficacy and safety, prolong celastrol biodistribution, reduce side effects of celastrol, such as liposomes, polymeric micelles, nanoparticles, micromulsions, inorganic and some other drug delivery system (Figure 8). The researches of several nano/micro-systems used to deliver celastrol and co-deliver celastrol and therapeutic drugs are listed in Tables 3, 4, respectively.

**TABLE 3:** The nanoparticles formulations of celastrol.

Nanocarrier classification	Nanocarrier composition	Encapsulation method	Particle Size (nm)	Cell lines	Cancer type	Feature	Ref
Carrier-free	—	Physical encapsulation	125.7	MCF-7/MDR	Breast cancer	Overcoming drug resistance	(Xiao et al., 2018)
Exosomal	Exosomes (isolated from bovine raw milk)	Physical encapsulation	106 ± 9	A549 and H1299	Lung cancer	Enhance its efficacy and reduce dose related toxicity	(Aqil et al., 2016)
Liposomes	Phospholipid, cholesterol, tween-80	Physical encapsulation	89.6 ± 7.3	Lewis cells	Lung carcinoma	Improve effective permeability; inhibit the tumor growth	(Song et al., 2011)
Liposomes	SPC, sodium deoxycholate	Physical encapsulation	128.1 ± 39.5	U251, C6 and SHG44	Glioma	Increase the bioavailability and reduce the side effects	(Huang et al., 2012)
Liposomes	Gala-PEG-DSPE, SPC, cholesterol	Physical encapsulation	139.4 ± 2.7	HepG2	Liver cancer	Improve the water solubility; enhance the therapeutic effect and reduce its adverse effects	(Chen et al., 2020)
Polymeric micelles	PEG-b-PCL	Physical encapsulation	48	SO-Rb 50	Retinoblastoma	Improve the hydrophilicity; inhibit the growth and induce apoptosis	(Li, Wu, et al., 2012)
Polymeric micelles	CTTP-CSOSA, NH_2_-PEG2000-NH_2_	Physical encapsulation	63.5 ± 18.0	MCF-7	Breast cancer	pH-sensitive; mitochondrial targeting	(Tan et al., 2018)
Polymeric micelles	TET-CSOSA, NH_2_-PEG2000-NH_2_	Physical encapsulation	82.5 ± 3.6	4T1	Breast cancer	avb3- targeted; improve antitumor metastasis therapy	(Zhao et al., 2018)
Phospholipid complex	SPC, PEG 400	Physical encapsulation	178.4 ± 7.07	—	—	Improve solubility and oral bioavailability	(Freag, Saleh, and Abdallah 2018b)
Phospholipid complex	CS, HPMC, protamine, SPC	Physical encapsulation	180.4 ± 6.16	—	—	Improve the absorption; higher bioavailability	(Freag, Saleh, and Abdallah 2018a)
Nanoparticles	PCL, tween 80	Physical encapsulation	75.4	LNCaP	Prostatic cancer	Improve the pharmacokinetics and biodistribution	(Yin et al., 2017)
Nanoparticles	SPC, TPGS, F68, IPM, glyceryl behenate	Physical encapsulation	90.2 ± 9.7	B16BL6	Melanoma	Enhance the percutaneous penetration and antimelanoma efficacy	(Chen et al., 2012)
Nanoparticles	CPP, precirol ATO-5, 1944CS, F68, TPGS, soybean lecithin	Physical encapsulation	126.7 ± 9.2	PC-3 and RM-1	Prostate cancer	Improve the hydrophilicity; enhance antitumor activity *in vitro* and *in vivo*; No signifcant adverse effects	(Yuan et al., 2013)
Lipid nanospheres	Lecithin, sodium oleate, soybean oil	Physical encapsulation	150	—	—	Enhance the oral bioavailability	(Zhang et al., 2014)
Nanoparticles	PCL, F-127	Physical encapsulation	175.5 ± 4.7	LNCaP, DU-145 and PC3	Prostate cancer	Exhibit remarkable antiproliferative activities	(Sanna et al., 2015)
Nanoparticles	PEG-PLGA, neutrophil membrane	Physical encapsulation	167.4 ± 2.6	Panc02	Pancreatic carcinoma	Enhance tumor inhibition which significantly prolonging the survival of tumor bearing mice and minimizing liver metastases	(Cao et al., 2019)
Nanoparticles	SF	Physical encapsulation	292.7 ± 28.1	—	Pancreatic ductal adenocarcinoma	Improve pharmacokinetic properties	(Onyeabor et al., 2019)
Nanoparticles	PEG-PLGA, neutrophil membranes	Physical encapsulation	—	B16F10	Melanoma	Neutrophils-targeted; prolong blood circulation; improve antitumor efficacy	(Zhou et al., 2019)
Inorganic systems	Glucose, PEI, MSN	Physical encapsulation	615	HeLa and A549	Cervical cancer; Lung cancer	Glucose-targeted; enhance anti-cancer activity; Did not induce any toxicity	(Niemela et al., 2015)
Inorganic systems	TiO_2_	Physical encapsulation	width about 80 nm and length range from 200 to 5,000 nm	HepG2	Liver cancer	Enhance the cytotoxicity of celastrol; reduce the side-effect	(Li et al., 2011)
Dendrimers	G5 PAMAM, PEG	Chemical conjugation	40	SW620	Colorectal cancer	Aptamers-targeted; reduce the side-effect	(Ge et al., 2020)

SPC, soybean phosphatidylcholine; gala-PEG-DSPE, galactose-modified 1,2-distearoyl-*sn*-glycero-3-phosphoethanolamine-poly(ethylene glycol); PCL, poly-(ε-caprolactone); PEG-b-PCL, poly(ethylene glycol)-block-poly(ε-caprolactone); CTPP, (4-Carboxybutyl) triphenylphosphonium bromide; SA, stearic acid; CSO, chitosan oligosaccharide; CS, laminated chitosan; HPMC, hydroxypropyl methylcellulose; TPGS, d-α-tocopherol polyethylene glycol succinate 1000; IPM, isopropyl myristate; CPP, cell-penetrating peptides; 1944CS, labrafil^®^ M 1944CS; F68, Pluronic F68; SF, silk fibroin; PEI, poly(ethylene imine); MSNs, mesoporous silica nanoparticles; TiO_2_, titanium dioxide; PAMAM, hydroxyl terminus poly(amidoamine).

**TABLE 4:** The anticancer nano/micro-systems for celastrol and therapeutic drugs co-delivery.

Nanocarrier classification	Nanocarrier composition	Combination drug	Encapsulation method	Particle Size (nm)	Cell lines	Cancer type	Feature	Ref
Liposomes	Coix oil, RH40, PEG400, SPC, cholesterol	STS	Physical encapsulation	94.8 ± 3.6	MCF-7	Breast cancer	Sequential drug release; Display diminished systemic toxicity	(Qu et al., 2018)
Liposomes	DPPC, SPC, GNR-DSPE-PEG, cholesterol	STS	Physical encapsulation	122.6 ± 0.6	MCF-7	Breast cancer	Photothermal-triggered; sequential drug release; display diminished systemic toxicity	(Qin et al., 2020)
Polymeric micelles	Celastrol-PEG-G Rh2	Ginsenoside Rh2	Chemical conjugation	121.53 ± 2.35	A549	Lung cancer	pH-sensitive; precisely release anticancer drugs and improve synergistic anti-lung cancer effect	(Li et al., 2017)
Nanoparticles	AEAA-PEG-BAP	Mitoxantrone	Physical encapsulation	112 ± 6	BPD6 and D4M	Desmoplastic melanoma	pH-sensitive and reduction-sensitive; reduce drug exposure and side-effects	(Liu et al., 2018)
Nanoparticles	SF	Triptolide	Physical encapsulation	TP: 166.4 ± 4.6 CL: 170.4 ± 2.3	MIA PaCa-2 and PANC-1	Pancreatic cancer	Increase the growth inhibition	(Ding et al., 2017)
Nanoparticles	HA, BSA, soybean oil	MT	Physical encapsulation	205.7 ± 5.4	Panc02	Pancreatic cancer	CD44 targeted; enhance tumor inhibition; alleviate the adverse effect and improve the safety	(Hu et al., 2019)
Microemulsions	DSPE-PEG-Tf, PEG 400, 1944CS, HS15	β-elemene	Physical encapsulation	69.2 ± 3.3	A549	Lung cancer	TF-targeted; exhibit enhanced antitumour activity; did not cause the obvious systemic toxicity	(Zhang Q. et al., 2019)
Microemulsions	TF, PEG 400	Coix seed oil	Physical encapsulation	27.7	HeLa	Cervical cancer	TF-targeted, enhance tumortargeting; facilitate deep penetration of drugs; enhance antitumor efficacy with little toxicity	(Guo et al., 2019)
Composite nanoparticles (MSN + Lipid)	MSN, cholesterol B, NBD-PC, DPPC, DSPE-PEG2000	Axitinib	Physical encapsulation	120	SCC-7, BT-474 and SH-SY5Y	Squamous carcinoma, breast cancer and neuroblastoma	Enhance antitumor efficacy	(Choi et al., 2016)

SPC, soybean phosphatidylcholine; STS, sodium tanshinone IIA sulfonate; DPPC, 1,2-Dipalmitoyl-sn-glycero-3-phosphocholine; GNR-DSPE-PEG, gold nanorods-1,2-distearoyl-sn-glycero-3-phosphoethanolamineN-[amino (polyethylene glycol); PEG, poly(ethylene glycol); G Rh2, ginsenoside Rh2; BAP, bis(acryloyloxymethyl)propionate; AEAA, aminoethylanisamide; SF, silk fibroin; HA, hyaluronic acid; BSA, bovine serum albumin; MT, 1-methyltryptophan; DSPE-PEG-Tf, transferrinmodified 1,2-distearoyl-sn-glycero-3-phosphoethanolamineN-[amino (polyethylene glycol); 1944CS, labrafil^®^ M 1944CS; HS15, kolliphor^®^ HS15; TF, transferrin; MSNs, mesoporous silica nanoparticles; NBD-PC, 1-palmitoyl-2-(6-[(7-nitro-2-1,3-benzoxa diazol-4-yl) amino]hexanoyl)-sn-glycero-3-phosphocholine.

#### Liposomes

**FIGURE 9:**
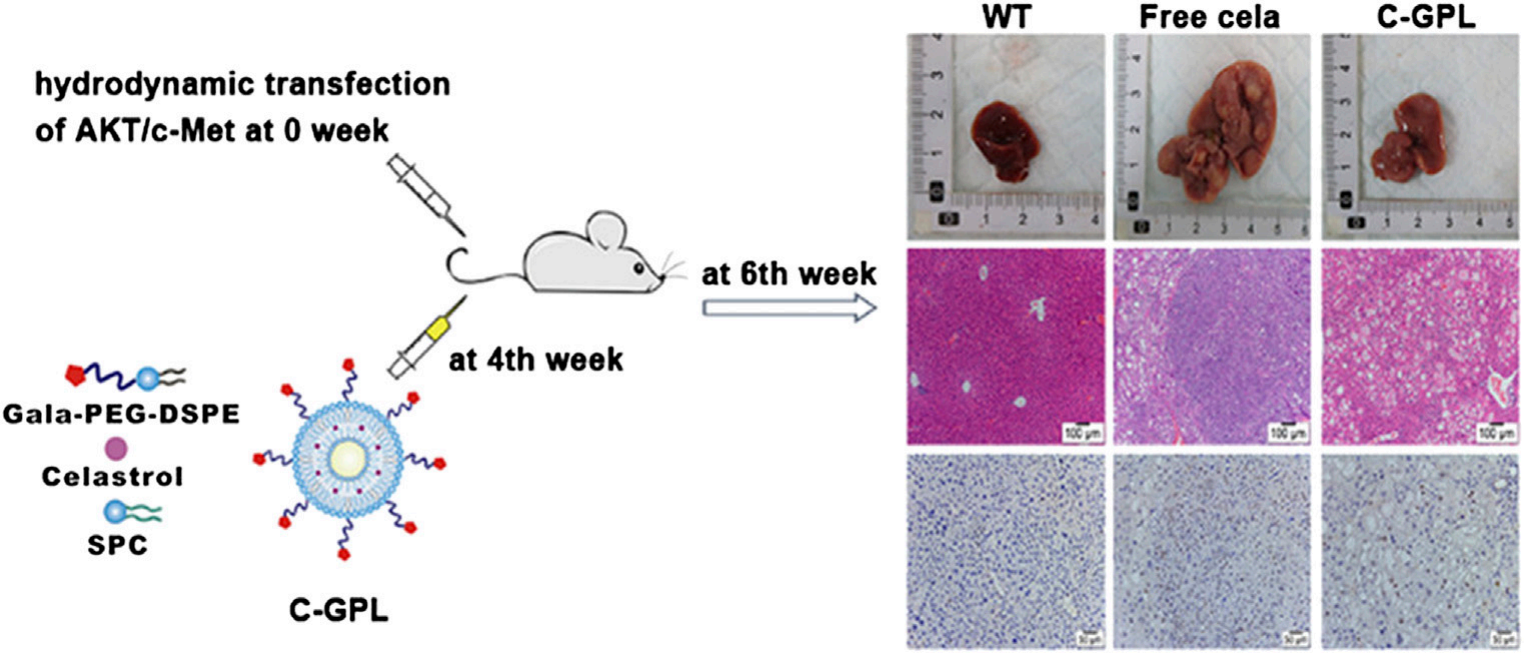
Liver-targeting liposomes were developed using galactose-modified 1,2-distearoyl-sn-glycero-3-phosphoethanolamine-poly(ethylene glycol) (gala-PEG-DSPE), natural soybean phosphatidylcholine (SPC), and cholesterol for delivery of celastrol to hepatic tissues (C-GPL). Reprinted with permission (Chen et al., 2020).

These vesicles are formed by a concentric lipid bilayer that entraps an aqueous core. The lipid membrane can be formed with phospholipids, lecithin and/or cholesterol and hydrophobic drugs can be incorporated in this bilayer, whereas hydrophilic drugs can be loaded in the aqueous core (Cagel et al., 2017). In one study (Song et al., 2011), celastrol-liposomes were prepared by the ethanol-injection method, and composed of phospholipid, cholesterol and Tween-80. The celastrol-loaded liposomes had improved effective permeability compared to the free drug in four intestinal segments. On the other hand, it also inhibited the tumor growth in C57BL/6 mice. Similarly, Huang et al. (2012) prepared liposomal celastrol using the thin-film dispersion method. In addition, a 4 mg/kg dose of liposomal celastrol had fewer severe side effects than free celastrol at the same dose. In this study, we found that the use of liposomes as a carrier of celastrol increased the bioavailability and reduced the side effects of celastrol. To overcome the shortcomings of celastrol and optimize its antitumor efficacy, galactose-modified PEGylated liposomes for targeted delivery of celastrol were prepared (Chen et al., 2020) (Figure 9). It could improve the water solubility of celastrol and exhibit high encapsulation efficiency, good stability in serum, and slow drug release profile. More importantly, it did not lead to serious weight loss and toxicity to normal organs.

On the other hand, a co-delivery system that sequentially releases its contents is an effective strategy to enhance anticancer efficacy. Researchers fabricated multicomponent-based liposomes loaded with sodium tanshinone IIA sulfonate (STS) and a small-sized microemulsion of celastrol, which shows synergistic anti-breast cancer activity through the initial release of STS for modulation of the tumor microenvironment, and subsequent release of celastrol for eradication of tumor tissues (Qu et al., 2018). Furthermore, it displayed diminished systemic toxicity compared to celastrol used alone. Thus offers a novel strategy for combination anticancer treatment and holds promising potential not only for breast cancer treatment, but also for the treatment of other solid tumors. Similarly, Qin et al. fabricate a gold nanorod-anchored thermo-sensitive liposomal complex co-loaded with STS and celastrol (G-T/C-L), which can sequentially release STS and celastrol upon NIR irradiation at 808 nm. When G-T/C-L reaches the sites, NIR illumination produces mild heat (∼43°C) and thereby triggers a rapid release of STS in the initial stage, decreasing the level of tumoral blood vessels, collagen, cancerassociated fibroblasts, and Th2 type cytokines. In the subsequent stage, celastrol was unloaded to exert anticancer effect under an activated tumor microenvironment. Due to the treatment of G-T/C-L with NIR illumination shows a significant improvement in anticancer efficacy both *in vitro* and *in vivo* but without conventional photothermal therapy-associated side effects, it enriches the application with combinational STS and celastrol in anti-breast cancer therapy.

#### Polymeric Micelles

**FIGURE 10:**
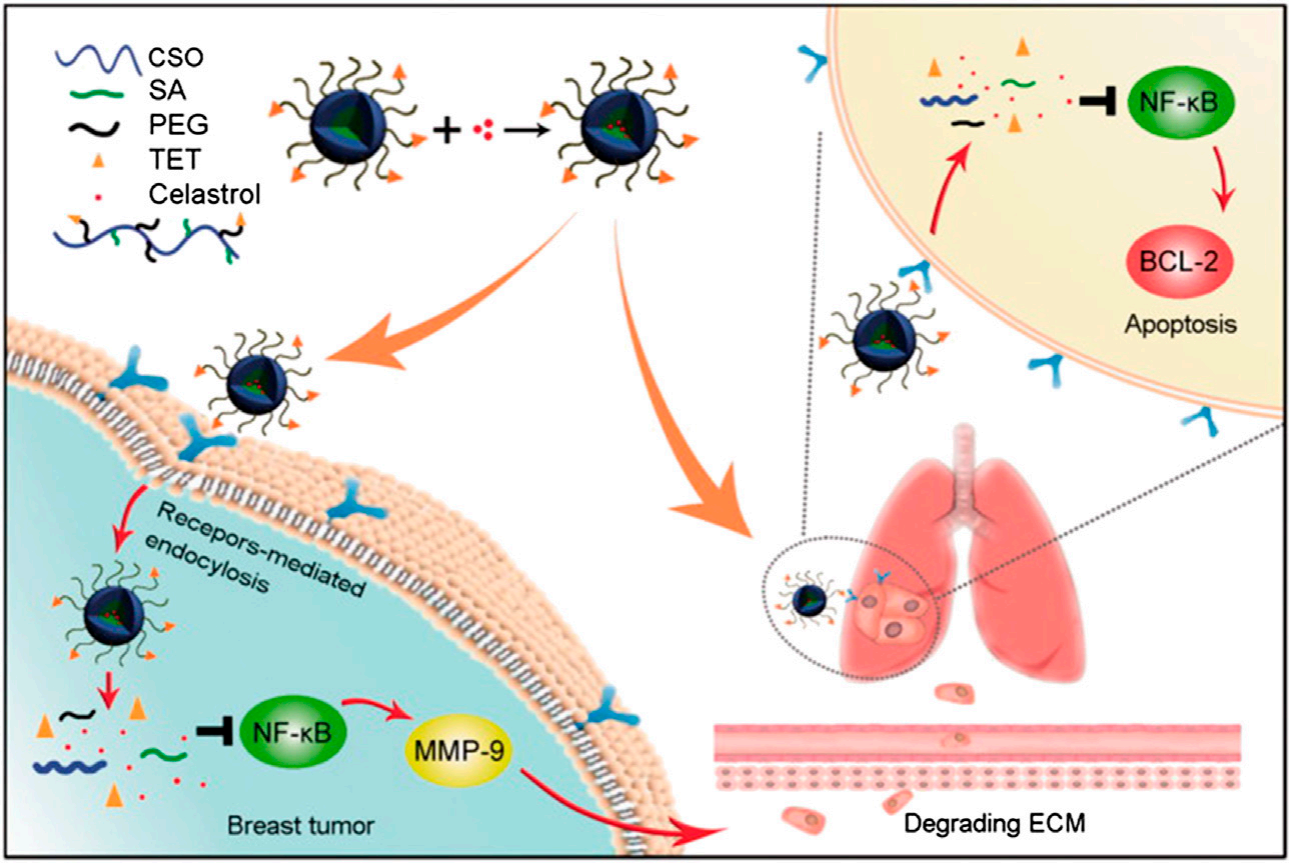
The schematic diagram of simultaneous targeting therapy for lung metastasis and breast tumor. Reprinted with permission (Zhao et al., 2018).

Li Z. et al. (2012), Li Y. et al., (2012) found that PEG-b-PCL micelles had promising potential to improve the hydrophilicity of celastrol and extend its release. Therefore, celastrol-loaded poly(ethylene glycol)-block-poly(ε-caprolactone) (PEG-b-PCL) nanopolymeric micelles were prepared to inhibit the growth of retinoblastoma and induce apoptosis in retinoblastoma cells in mice. Active targeting therapy-mediated drug delivery system has been shown to reduce systemic toxicity and achieve targeted synergistic effects (Mo et al., 2014; Wang et al., 2014). A celastrol loaded glucolipid-like conjugates (CSOSA/Cela) with avb3-ligand tetraiodothyroacetic acid (TET) modification (TET-CSOSA/Cela) were established to preparation of polymer micelles. In this study, it exploited a delivery system for improved antitumor metastasis therapy, which not only targeted breast tumor but also lung metastasis by means of avb3 receptor-mediated interaction. The results of 4T1 metastasis inhibition showed that TET-CSOSA/Cela could suppress breast tumor invasion and lung metastasis growth through inhibition of NF-κB signaling pathway (Figure 10) (Zhao et al., 2018). In another study, the (4-Carboxybutyl) triphenylphosphonium bromide (CTTP)-CSOSA/Cela micelles were developed for mitochondrial targeting and alkaline pH-responsive drug release to treat cancer. Importantly, CTPP-CSOSA/Cela micelles could selectively accumulate in tumor cell mitochondria, and realize fast drug release by responsing to mitochondrial alkaline pH environment, resulting in the significant apoptosis of tumor cells. Furthermore, CTPP modified CSOSA gave rise to its accumulation in tumor tissues (Tan et al., 2018). Li et al. (2017) report the development of a polymeric material with a three-section structure, “hydrophobic-hydrophilic-hydrophobic,” through the covalent conjugation of celastrol and ginsenoside Rh2 onto both ends of PEG segments via ester linkages. It could rapidly release drugs under acidic and enzymatic conditions, but slowly released in normal physiological environments. Therefore, it is a promising vector for precisely releasing anticancer drugs within the tumor cells, and thereby exerts an improved synergistic anti-lung cancer effect.

#### Nanoparticles

Oral delivery of celastrol remains challenging because of limited water-solubility and/or poor permeability. Nanostructured lipid carriers (NLCs) are attractive materials for topical drug delivery. The surface charge of NLCs has a great inﬂuence on the skin permeation and pharmacodynamics of celastrol. Cationic celastrol-loaded NLCs could enhance the percutaneous penetration and antimelanoma efficacy of celastrol and offer several advantages over celastrol alone (Chen et al., 2012). Similarly, aimed to enhance the oral bioavailability of celastrol, celastrol-loaded lipid nanospheres (LNs) were prepared by rapid dispersion of an ethanol mixture of celastrol, lecithin, sodium oleate, and soybean oil into water. The pharmacokinetic results showed that LNs significantly enhanced the oral bioavailability of celastrol with a relative bioavailability of 224.88% (celastrol suspensions was used as a reference). The mechanistic studies demonstrated that improved intestinal permeability and post-enterocyte lymphatic transport were mainly responsible for the enhanced oral absorption (Zhang et al., 2014).

Sanna and colleagues (Sanna et al., 2015) indicated that nanoencapsulation would represent a powerful strategy to overcome these issues of celastrol. In their study, they developed novel celastrol-loaded PCL nanoparticles, and the nanoparticles significantly increased cytotoxicity at lower/medium dose (0.5 and 1.0 µM) on DU145 and PC3 cell lines with respect to free celastrol. Besides, to improve the hydrophilicity of celastrol, Chen and co-workers developed cell-penetrating peptides (CPP)-coated celastrol-loaded NLCs. It noticeably enhanced antitumor activity *in vitro* and *in vivo* with no significant adverse effects (Yuan et al., 2013). Additionally, due to the application of celastrol-loaded silk fibroin (SF) nanoparticles, the pharmacokinetic profile was improved with celastrol-loaded SF nanoparticles compared to pure celastrol-which was observed following IV administration. The initial concentration of celastrol nanoparticles was four times higher than that of celastrol in solution and celastrol-SF nanoparticles demonstrated longer mean residence time (Onyeabor et al., 2019). In another study, researchers aimed to develop polymeric nanoparticles combined with the reticuloendothelial system (RES) saturation to improve the *in vivo* distribution and antitumor activity of celastrol. The pharmacokinetic studies revealed that celastrol-nanoparticles had the advantage in bettering the pharmacokinetic properties of celastrol over the solution formulation. However, the ameliorative effect on pharmacokinetics was more significant in the case of RES saturation (Yin et al., 2017).

Recently, the application of neutrophils as therapeutic vehicles in cancer therapy has gradually been recognized (Xue et al., 2017). Meanwhile, Kang et al. (2017), Li R. et al. (2018) highlighted the use of activated neutrophil membrane-coated nanoparticles targeting to circulating tumor cells in the treatment of metastatic breast tumor. Hence, Cao et al. (2019) fabricated naïve neutrophil membrane-coated PEG-PLGA nanoparticles (NNPs) to achieve pancreas-specific delivery of celastrol by overcoming the blood-pancreas barrier. The result showed NNPs selective accumulations at the tumor site following systemic administration as compared to nanoparticles without neutrophil membrane coating. In both orthotopic and ectopic tumor models, celastrol-loaded NNPs demonstrated greatly enhanced tumor inhibition which significantly prolonged the survival of tumor bearing mice and minimizing liver metastases. Similarly, celastrol-loaded PEG-PLGA nanoparticles coated with neutrophil membranes displayed significantly enhanced cytotoxicity and apoptosis rate in a murine melanoma cell line B16F10 compared to celastrol-loaded PEG-PLGA nanoparticles (Zhou et al., 2019).

**FIGURE 11:**
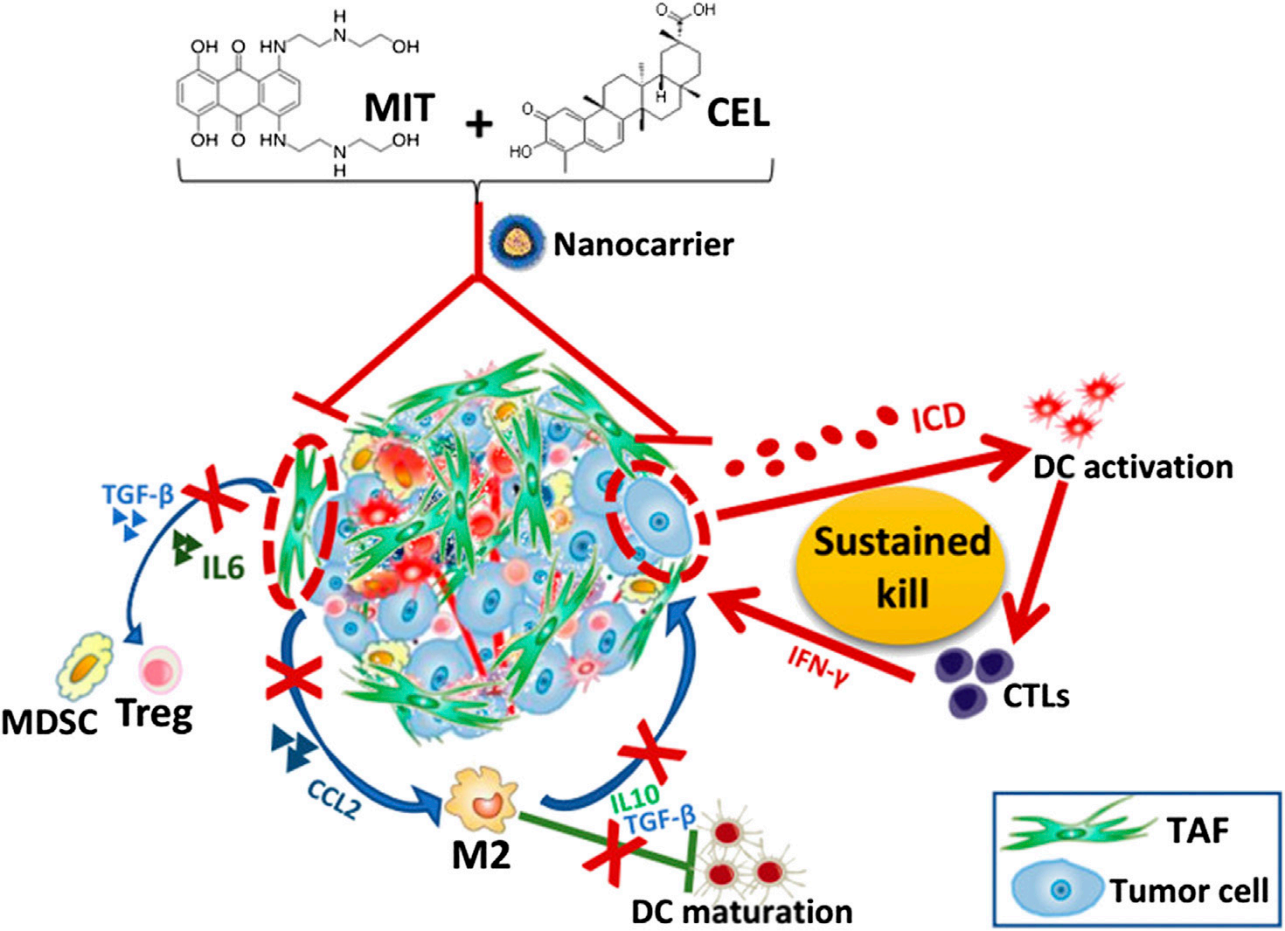
Figure legend depicting nanocarrier-mediated chemo-immuno therapy by co-delivering MIT and CEL. MIT, mitoxantrone; CEL, celastrol; TAF, tumor associated fibroblast; ICD, immunogenic cell death; DC, dendritic cell; CTL, cytotoxic T lymphocyte; MDSC, myeloid-derived suppressor cell; Treg, regulatory T cell; M2, M2 macrophage. Reprinted with permission (Liu Q. et al., 2018).

Combination therapy is the routine strategy of cancer chemotherapy with significant advantages including lower treatment failure rate and slower development of drug resistance. Ding and coworkers (Ding et al., 2017) developed and characterized triptolide-loaded SF nanoparticles and celastrol-loaded SF nanoparticles separately, which not only overcame the pitfall of hydrophobicity, but also facilitated triptolide and celastrol passively accumulating in cancerous tissues based on the Enhanced Permeability and Retention effect as well as definition of optimal dose and schedule of administration. Further, hierarchical assembly of hyaluronic acid coated albumin nanoparticles were utilized to co-deliver celastrol and 1-methyltryptophan (MT). The nanoparticles with a unique hollow structure selectively accumulated in both the xenograft pancreatic tumor and the orthotopic pancreatic tumor site following systemic administration possibly via size-reduction effect, and the CD44 mediated accumulation and internalization. Thus significantly enhanced tumor inhibition and alleviated the adverse effect and improved the safety of using celastrol *in vivo* (Hu et al., 2019). Another study showed that developed an innovative chemo-immuno strategy based on targeted delivery of mitoxantrone and celastrol, two potent medicines screened and selected with the best anti-cancer and anti-fibrosis potentials. The combination targets tumor-associated fibroblasts to reduce the desmoplasia of the tumor. The chemo-immuno therapy significantly remodeled immune-suppressive tumor microenvironment, as well as triggered a robust immune memory response. Since only low doses of both drugs were used, the treatment was without any toxicity to the host (Liu Q. et al., 2018) (Figure 11).

#### Microemulsions

Microemulsions result from the dispersion of two immiscible liquids, typically water and oil, and are stabilized using an appropriate surfactant (Singh et al., 2017). It has been reported that transferrin-functionalized microemulsions coloaded with coix seed oil and celastrol (Tf-CT-MEs) for the treatment of cervical cancer, which promoted accumulation at the tumor site, improved permeability in tumor tissues, retarded tumor growth, inhibited tumor cell proliferation, promoted tumor cell apoptosis, enhanced antiangiogenesis, and downregulated the concentration of protumoral cytokines in serum. Meanwhile, Tf-CTMEs showed little toxicity against normal noncancerous organs (Guo et al., 2019). Using tumor-targeted and combinational microemulsions, this study provides a potential strategy for improving anticervical cancer therapy. In another study, co-delivery of β-elemene and celastrol using Tf-functionalized microemulsion (Tf-EC-MEs) exhibited enhanced antitumor activity compared to all other treatments. More importantly, Tf-EC-MEs did not cause the obvious systemic toxicity commonly found with mono-celastrol treatment (Zhang Q. et al., 2019). Therefore, using tumor-targeted and combinational microemulsions provides a potential strategy for improving anti-cancer therapy of celastrol.

#### Inorganic Nano-Systems

Li et al. (2011) introduced biocompatible titanium dioxide (TiO_2_) nanofibers into the research of celastrol, And after the TiO_2_ nanofibers were introduced into the system of celastrol, the cooperation effect showed that the nanocomposites between TiO_2_ nanofibers and celastrol could enhance the cytotoxicity of celastrol for HepG2 cells and cut down the drug consumption so as to reduce the side-effect of celastrol. In addition, Niemelä and colleagues (Niemela et al., 2015) utilized glucose as an affinity ligand decorated on mesoporous silica nanoparticles (MSNs), with the aim of delivering these celastrol-loaded MSNs with high specificity to cancer cells and inducing minimal off-target effects in healthy cells. MSNs were thus functionalized with sugar moieties by two different routes, either by conjugation directly to the MSN surface or mediated by a hyperbranched poly (ethylene imine, PEI) layer; the latter to increase the cellular uptake by providing an overall positive surface charge as well as to increase the reaction sites for sugar conjugation. The particles themselves did not induce any toxicity, and normal cells displayed minimal off-target effects. During the study carried out by Choi and teams (Choi et al., 2016) the researchers prepared axitinib/celastrol-loaded combination nanoparticles (ACML) with celastrol loaded in the MSN and axitinib in PEGylated lipidic bilayers. It demonstrated that the administration of ACML effectively inhibited angiogenesis and mitochondrial function and was efficiently internalized in the SCC-7, BT-474, and SH-SY5Y cell lines.

#### Other Nano-Systems

Xiao et al. (2018) designed and synthesized a carrier-free and biocompatible nanomedicine based on a simple and green self-assembly method, for synergistic combination chemotherapy of celastrol and doxorubicin to overcome MDR and promote chemotherapeutic effect. Spherical nanoparticles can improve the water-solubility of celastrol, reduce the dosage of doxorubicin, and therefore obviously enhance cellular drug accumulation via activating Heat shock factor 1 (HSF-1) and inhibiting NF-κB to depress P-gp expression, which result in apoptosis and autophagy of doxorubicin resistant cells through ROS/JNK signaling pathway. Aqil et al. (2016) isolated exosomes from bovine raw milk, and used physical encapsulation to prepare celastrol-loaded exosomes. The data have shown that celastrol-loaded exosomes enhance free celastrol efficacy and reduce dose-related toxicity in lung cancer. Exosomes loaded with celastrol exhibited enhanced anti-tumor efficacy as compared to free celastrol against lung cancer cell xenograft. Besides, celastrol did not exhibit any gross or systemic toxicity in wild-type C57BL6 mice as determined by hematological and liver and kidney function test. Freag and coworkers developed self-assembled phytosomal nanocarriers for improving celastrol solubility and oral bioavailability. Celastrol-phospholipid complex (CST-PHY) was prepared using simple solvent evaporation technique. The pharmacokinetic studies in rabbits revealed significant improvement in CST-PHY oral bioavailability compared with crude celastrol evidenced by 4-fold increase in AUC_0-8_ and 5-fold increase in C_max_ of CST-PHY compared with crude celastrol (Freag et al., 2018b). The teams also reported (Freag et al., 2018a) to surmount the celastrol’s pharmaceutical obstacles and improve its bioavailability, protamine-coated phytosomal nanocarriers were prepared. And, the nanocarriers was loaded into laminated chitosan (CS): hydroxypropyl methylcellulose (HPMC) composite sponges as flexible and soft mucoadhesive dosage form to control drug release kinetics and permeation via buccal mucosa. Conclusively, mucoadhesive CS-HPMC sponges loaded with a novel mucopenetrating nanocarrier could significantly improve the absorption of celastrol via buccal mucosa which would be of prime importance for its clinical utility. In another team (Ge et al., 2020), their work aimed at developing bioconjugates composed of EpCAM aptamer, PEG and dendrimers for specifically delivering celastrol into EpCAM-abundant tumors to improve the antitumor efficacy and mitigate the toxicity. The results exhibited much reduced local and systemic toxicity both in xenograft mice and zebrafish models. Thus these data indicate that the integrated strategy-designed dendrimer delivery system represents a promising application of celastrol in targeted cancer treatment with great biosafety and specificity.

## Current Perspectives and Challenges

### Taking Advantage of Combination Chemotherapy

Effective combination therapy for anticancer treatment requires dealing with an investigation of multiple hurdles. Moreover, prior to selecting the combination of therapeutic agents, a deep analysis of cancer pathways involved, feedback loops, alternative mechanisms, genetic profile and existed treatment obstacle should be considered thoroughly. Due to the important role of celastrol in the EMT, the design of combination strategies can be based on this to carry out more research. Moreover, tumor microenvironment favors tumor cells to promote their growth and metastasis such as migration, invasion, and angiogenesis. Lee et al. introduce celastrol, as an inhibitor of NLRP3 infammasome, to reduce the potency of macrophages to stimulate migration and invasion of melanoma cells, which provide a novel anti-cancer strategy to modulate tumor microenvironment by suppressing NLRP3 infammasome and consequently reducing IL-1β production (Lee et al., 2019). Therefore, combining drug strategy based on tumor microenvironment may achieve twice the result with half the effort. More systematic and mechanistic studies are useful to identify optimal drug synergism, and intensification of research on combination regimes with other therapies in clinical use could facilitate translation of celastrol formulations (Baran et al., 2014; Mahbub et al., 2015). On the other hand, in addition to conventional chemotherapy, we need as many combinations of modern therapies as possible, such as gene therapy, immunotherapy, photothermal or photodynamic therapy, etc. (Liu Y. et al., 2018; Haasteren et al., 2018; Sanmamed and Chen, 2018; Sun et al., 2019).

### Taking Advantage of Analogs of Celastrol

Although celastrol has already been proved to have significant anticancer activity through functional phenotypic screening *in vitro* and *in vivo*, its precise molecular targets that responsible for the potent biological activity have not yet been fully identified (Kashyap et al., 2018). Hence, it is important to further design and synthesis of new bioactive probes of celastrol to identify unexplored molecular targets and map the integral signaling networks that responsible for its effects and toxicity.

The reported modifications are limited on C-2, C-3 of A-ring, C-6 of B-ring and C-20 of E-ring, while decoration on other sites of this molecule is still underdeveloped. Therefore, despite it is of great challenging, a part of the future work should be devoted to investigating the structure-activity relationship caused by modification on other sites of celastrol.

Biotransformation or biosynthesis is becoming an increasingly powerful tool for the synthesis or structure modification of complex natural products (Jobby et al., 2018; Khojasteh et al., 2018). Therefore, it is meaningful to explore key enzymes or organisms that are responsible for the synthesis of key intermediates of celastrol or site-specific functionalization of celastrol.

### Taking Advantage of Nano/micro-systems

An ideal drug delivery system maintains the drug within a desired therapeutic range after a single dose, and/or target the drug to a specific region while simultaneously lowering the systemic levels of the drug. Many diﬀerent systems and strategies have been evaluated for drug targeting to tumors over the years. In order to prolong blood circulation, “Stealth” systems of PEG is the most effective approach to achieve stability and improve long-circulation effect (Chen et al., 2016). In addition, the nanocarriers can also be used to deliver drugs through surface modification by binding the target molecules to the highly expressed receptors on the surface of tumor cells (Kim et al., 2019). It selectively recognizes specific molecules in disease tissues and improve therapeutic index while reducing serious side eﬀects on normal healthy tissues. Common targeted modification molecules include folic acid (Prasad et al., 2018), hyaluronic acid (Huang and Huang, 2018), peptide (Xiao et al., 2019; Yi et al., 2020), transferrin (Zhang Y. et al., 2018), biotin (Li K. et al., 2019), etc.

In order to overcome delivery barriers, nanocarriers can be designed to vary with the tumor microenvironment, including charge reversal, shell detachment, size transition, etc (Sun et al., 2017). Moreover, by adjusting the release response mechanism and release rate of drugs under different stimuli, the nanocarriers can control the sequence and time schedule of drug combination therapy, so as to achieve more accurate drug delivery process and improve the effect and specificity of combined action (Zhu et al., 2017). The drug release behavior can be realized in different stimulant responses in tumor therapy, such as pH (Wang et al., 2017), hypoxia (Kumari et al., 2020), reduction (Xie and Liu, 2020), enzyme (Shan et al., 2019), temperature (Jommanee et al., 2018), ultrasound (Xia et al., 2018), etc.

Endogenous nanocarriers, as compared with synthetic nanoformulations, have shown promising results in enhancing drug delivery and therapeutic efficacy because of their native biocompatibility *in vivo* (Batrakova and Kim, 2015). For example, exosomes are emerging as an effective therapeutic tool for various pathologies, which cannot be treated by conventional medicine. These sub-micron-sized vesicles are secreted by different cell types and participate in intercellular communication by fusing with the recipient cell membrane and thus delivering their payload (genetic information and proteins) to the cell (Betzer et al., 2017). In addition, some exosomes also exhibit an increased capacity to escape degradation or clearance by the immune system (Luan et al., 2017). Therefore, exosomes are ideal natural nanocarriers for clinical application of celastrol because of their naturally biocompatible characteristics.

Despite many developments on multifunctional theranostic nano-systems for diagnostic imaging and tumor therapy, we need pay more attention to the attempts on the design of ‘self-reporter nano-systems’ which can not only be used for drug delivery, but also as a real-time feedback of *in vivo* tumor response to treatment.

## Conclusion

Natural products continue to serve as an important and invaluable source of new drug discovery. Celastrol is one of the most potent chemotherapeutic agents effective against a variety of cancers including breast, liver, lung and ovarian cancer. The poor physicochemical characteristics of the celastrol are major challenges in the formulation development and as a result there are very limited formulation options in clinic for this drug. Besides, the used celastrol which has been found to cause various adverse effects including infertility toxicity, cardiotoxicity, hepatotoxicity, hematopoietic system toxicity and nephrotoxicity due to weak targeting. Therefore, the researchers tried different strategies to overcome these obstacles. This review systematically summarizes the combination chemotherapy of celastrol in different cancers, including combination with chemotherapeutic agents, tumor necrosis factor superfamily, active ingredients of Traditional Chinese Medicine, IR and nucleic acid. Successful combinations can enhance the therapeutic efficiency of celastrol. Moreover, lowering the dosage used of celastrol by combining it with agents effectively reduces their related adverse effects. These results highlight it is beneficial to improve the deficiency of celastrol and expand its application range. Furthermore, structural modification could promote physical and chemical properties and pharmacokinetics by improving stability, solubility, selectivity and biological activity. Additionally, nano/micro-medicine formulations endeavor to improve both the toxicity profile and therapeutic efficacy relative to the conventional drug formulation. Nano/micro-systems encapsulated celastrol have been employed to improve celastrol aqueous solubility, chemical stability, efficacy and safety, prolong celastrol biodistribution, reduce side effects of celastrol.

Accordingly, the following three suggestions are proposed for further research on celastrol. 1) A complete pharmacokinetic profiling and in vitro-in vivo correlation for the drug combinations, needs to be drawn to further move up the ladder. 2) The development of celastrol-based drug combination would also be a useful strategy, such as the use of a protective agent to reduce its toxicity, the combination of celastrol with other anticancer agents to gain an increased anticancer activity to overcome the development of drug resistance. 3) The clinical translations of combination antitumor therapy and structural modification will be also investigated extensively, and the well-designed nanocarriers and nanoformulations with simple manufacturing process, good reproducibility and easy quality control will attract more attentions, which eventually lead to the rapid clinical translation of celastrol.

## Author Contributions

JS and JL wrote the first draft. JS, JL and ZX performed literature survey and data extraction. LC and RL provided the organization and framework of the article. CZ, FG, JZ and CF provided critical revisions. All authors approved the final version of the manuscript for submission.

## Funding

This research was supported by China Postdoctoral Science Foundation (2017M612930), Distinguished Young Science and Technology Talents of the Science and Technology Department of Sichuan Province (2019JDJQ0049) and National Natural Science Foundation of China (81973662).

## Conflict of Interest

The authors declare that the research was conducted in the absence of any commercial or financial relationships that could be construed as a potential conflict of interest.
